# Nanoengineered catalysts for sustainable pollutant removal from industrial effluents

**DOI:** 10.1186/s11671-026-04970-3

**Published:** 2026-10-05

**Authors:** Jibanjyoti Panda, S. Sowjanya, Bishwambhar Mishra, Satya Kumar Avula, Sarvesh Rustagi, Mrunal S. Wagh, Shibani Mohapatra, Alok Kumar Panda, Yugal Kishore Mohanta

**Affiliations:** 1 Centre for Industrial Biotechnology Research, Shiksha “O” Anusandhan (Deemed to be University), Bhubaneswar, 751029 Odisha India; 2 School of Biosciences and Technology, VIT School of Agricultural Innovations And Advanced Learning, Vellore Institute of Technology, Vellore, 632014 Tamil Nadu India; 3 Department of Biotechnology, Chaitanya Bharathi Institute of Technology (CBIT), Gandipet, Hyderabad, 500075 Telangana India; 4 Natural and Medical Sciences Research Centre, University of Nizwa, 616, Nizwa, Oman; 5 Department of Food Technology, School of Allied Sciences, Dev Bhoomi Uttarakhand University, Dehradun, Uttarakhand, 248007 India; 6 Research & Development Division, Organica Biotech Private Limited, Mumbai, 400088 Maharashtra India; 7 Centre for Biotechnology, Shiksha “O” Anusandhan (Deemed to be University), Bhubaneswar, 751029 Odisha India; 8 Environmental Science Laboratory, School of Applied Sciences, KIIT (Deemed to be University), Bhubaneswar, 751024 Odisha India; 9 Nano-biotechnology and Translational Knowledge Laboratory, Department of Applied Biology, University of Science and Technology Meghalaya, Techno City, 9th Mile, Baridua, Ri-Bhoi-793101, Meghalaya, India; 10 Centre for Herbal Pharmacology and Environmental Sustainability, Chettinad Hospital and Research Institute, Chettinad Academy of Research and Education, Kelambakkam, 603103 Tamil Nadu India

**Keywords:** Nanocatalysts, Pollutant degradation, Wastewater treatment. Heavy Metal Removal, Environmental Remediation

## Abstract

Our ecological balance is being disrupted by the growing population and industrial waste, which causes pollution and climate change. Our health is at risk from these toxins, which can lead to illnesses. These contaminants must degrade, but some are difficult to break down and take longer to remediate. To solve the urgent problem of environmental pollution, nanotechnology can assist in converting harmful toxins into innocuous ones or appropriately remediating them. This draws attention to the drawbacks of traditional wastewater treatment methods and the need for new technologies. Nano-engineered catalysts possess unique properties and a large surface area that provide greater reactivity and selectivity for the degradation of pollutants. This review will discuss several different categories of nano-catalysts, including metal-based nanoparticles (e.g., Fe_2_O_3_ and Ag-NPs) and polymeric nanomaterials, and examine their applications and performance in pollutant removal, with an emphasis on heavy metals, organic compounds, and dye removal. Discussing the combination of catalytic and biocatalytic processes to develop hybrid systems that are more effective in degrading and removing pollutants. Major developments in industrial wastewater treatment technologies, including hybrid, biocatalytic, and nano catalyst technologies, have been explored and investigated for their potential to improve pollutant degradation whilst preserving environmental sustainability. Long-term stability, cost-effectiveness, and scalability concerns are further addressed to open up novel opportunities for the study and use of nano-engineered catalysts in wastewater treatment.

**Graphical Abstract:**

**Figure:**
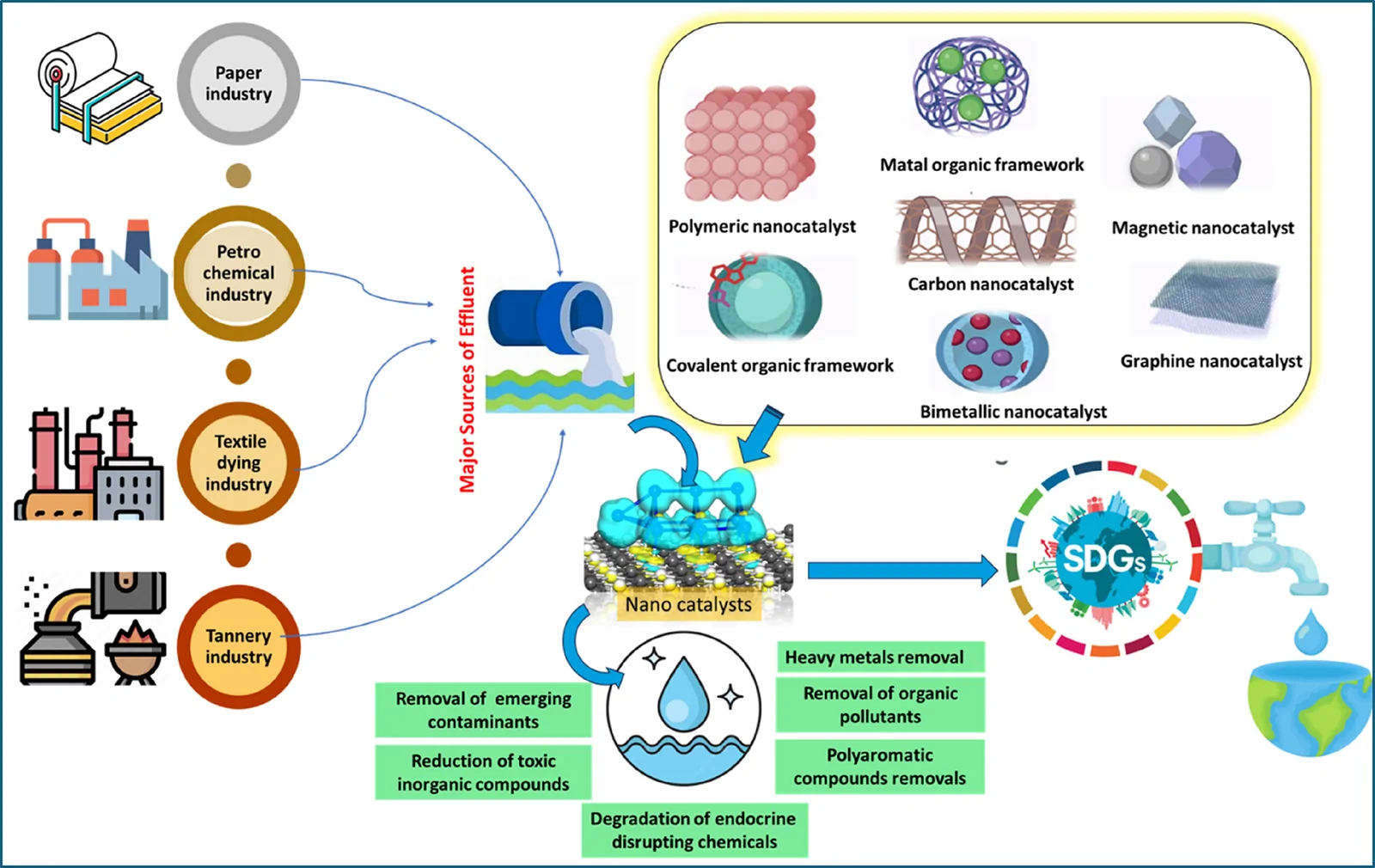

## Highlights

Nanocatalysts offer high reactivity and selectivity for breaking down stubborn pollutants.

Their large surface area enhances efficiency in removing heavy metals, dyes, and toxins.

Hybrid systems combining nanocatalysis and biocatalysis boost pollutant degradation.

## Introduction

In recent years, advances in technology have made living easier, but they also produce waste from modern businesses that pollute the environment. These leftovers harm the environment when they are released into the air, soil, water, and the natural ecosystem. These challenges can be addressed by bioremediation, which is made even better by the introduction of new technologies. Environmental pollution is caused by major contaminants like particulate matter, toxic metals, fertilisers, pesticides, herbicides, oil spills, poisonous gases, sewage, industrial effluents, and organic compounds [1]. To provide effective and affordable purifying treatment solutions, the scientific community must concentrate on wastewater recycling. To eliminate oxygen-demanding chemicals and inorganic pollutants, traditional techniques such as membrane filtration, activated sludge, and waste stabilisation ponds have been researched [2]. Nevertheless, dangerous pollutants have been found in treated industrial effluents, which represent a serious risk to the environment and necessitate additional research to overcome [3].

At the nexus of nanoscience and catalysis, nano-catalysis is a vibrant and revolutionary field that is at the front of scientific and industrial innovation. Fundamentally, nano catalysis entails catalytic reactions that take place at the nanoscale, where materials display distinct and frequently noteworthy characteristics [4]. Nanomaterials, complex nanoparticles or tiny atoms, have replaced conventional compounds due to their higher selectivity and reactivity than bulk materials. With their high density, significant surface area to volume ratio, and wide range of functions, these nanoparticles hold promise as a potential replacement for bulk materials [5]. Nano catalysts, which are often composed of metals, metal oxides, or organic substances, differ from their bulk counterparts due to their enormous surface area along with quantum size effects. Nano-catalysis significantly impacts current research and industry as it offers control over reaction paths and selectivity. It is essential for processes such as fine chemical synthesis and environmental cleanup, which are revolutionising energy conversion and storage technologies. Nano-catalysts can also be used to address global challenges like climate change and resource sustainability [6] .

Nanomaterials can be generally divided into three groups based on the materials that constitute them. Graphene, fullerene C540, fullerene C60, multi-walled nanotubes, single-walled nanotubes, and inorganic nanomaterials like metal and metal oxide are among them, as are polymer nanomaterials. Polymer-based nanomaterials are thought to be the most effective of these three for detecting and eliminating contaminating substances such as nitrate, heavy metals, iron, manganese, arsenic, etc. Additionally, it effectively separates gases like CO, SO_2_, and NOx [7, 8]. In this regard, to stop additional pollution from occurring from their remaining sources, the materials used for pollution remediation must be non-toxic. Biodegradable materials are ideal for use in nature. The usage of biodegradable materials is more beneficial since they are safer and may be employed in any circumstance, given that there is no recyclable material to be disposed of following treatment. Thus, the development and utilisation of nanomaterials have garnered an extensive amount of concern globally owing to their numerous enduring advantages [1].

Experts have been investigating the application of nanomaterials to purge polluted water sources of contaminants since the 1970s [9]. The broad adsorption surface, minimal temperature modification, active atomicity, and chemical reactivity of these materials make them promising adsorbents and catalysts. Surface atoms’ modest size enables them to adsorb and interact with other atoms, stabilising charges and lowering pollution levels. In addition to their effective adsorption capabilities, they also possess special redox characteristics that aid in the degradation of redox-sensitive contaminants [9]. Nevertheless, several widely utilised nanomaterials do have drawbacks, such as exorbitant prices, possible toxicity, recycling challenges, and simple media interaction. Even though nanoparticles have been studied extensively in the field of heavy metal remediation, there are few comprehensive and systematic evaluations of their application for the removal of heavy metal particles [10].

The central aim of this article is to present a detailed evaluation of the current state of industrial effluents, their impacts on the environment, and their requirements for effective pollutant removal technologies. This study aims to evaluate the role of catalysis in wastewater treatment and presents the production and application of nano-engineered catalysts within the context of this role. The review seeks to integrate the latest advancements in catalysts, biocatalytic, and hybrid technologies for the treatment of industrial wastewater, following a specific focus on their capabilities to improve the degradation of pollutants while maintaining sustainability standards.

Unlike other reviews that concentrate on one class of nanocatalyst or one class of treatment mechanism, the present reviews the state of the art in nano-engineered metal, carbon, bimetal, MOF/COF, and enzyme-mimetic catalysts and provides cross-comparative evaluation of the catalysts. Integrating the mechanistic studies with green and advanced synthesis, surface functionalization, and performance on target pollutants synthesized for the first time in major industrial waste streams of textile, paper, petrochemical, and tannery industrial waste, is a first of its kind effort. Additionally, linking the design of the catalysts to their scalability in the context of emerging contaminants, to the Techno-Economic challenges of the future, this review provides a much wider and application-based scope than any in the existing literature. These factors combine to demonstrate a clear gap and novel contribution of the review to the field of catalytic treatment of wastewater.

## Various sources of industrial effluents and their treatment

**Fig. 1:**
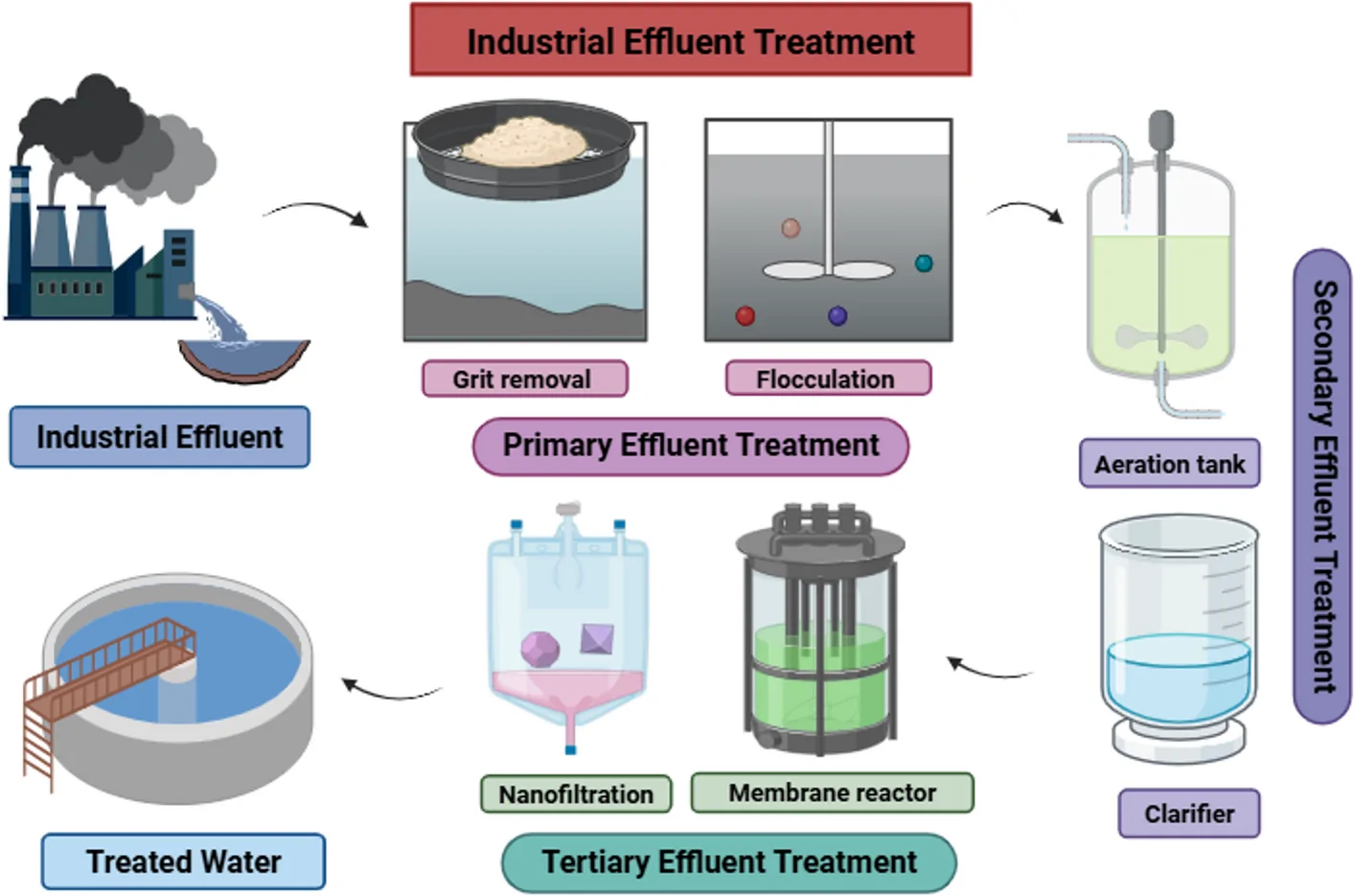
General effluent treatment methods

Industrial wastes discharge from varied industries including mining, electroplating, tanning, petrochemicals, textiles, and plastics which each discharge different types of dangerous pollutants to the environment. These pollutants encompass heavy metals like mercury, chromium, lead, and cadmium. Organic pollutants like phthalates, benzene derivatives, pesticides, and endocrine disrupting chemicals also severely threaten human and ecosystem health. Figure 1 depicts the general methods of effluent treatments while Table 1 lists the key pollutants and their health effects. Knowledge of the types and sources of these effluents is required to formulate effective, specific treatment technologies.

**Table 1:** Impact of industrial effluents and key Pollutants

Industrial Effluent	Major Pollutant	Environment and Health Impact	Reference
Industrial discharge, mining, fossil fuel combustion	Mercury	Cognitive and motor dysfunction, neurodevelopmental issues	[11]
Electroplating, leather tanning	Chromium	Respiratory issues, lung cancer, kidney damage	[12]
Wood digestion	Sulfur dioxide	Chronic bronchitis	[13]
Chrome Plating	Pb (II)	Damages to the central nervous system	[14]
Bleaching and use of preservatives	benzene - nitro and amino compounds of benzene and its homologues - nitrous oxide	Blood disorders	[13]
Leaded gasoline, industrial processes, lead pipes	Lead	Developmental delays, hypertension, renal impairment	[15]
Plastic manufacturing, plasticizers, flame retardants, stabilizers	Phthalates and Bisphenol A	Endocrine-disrupting chemicals	[16]
Pesticides	Organophosphate, carbamate	cancer, Parkinson’s, Alzheimer’s, DNA and RNA damage and problems with the reproductive system.	[17]
Mining, smelting, and electroplating	copper, iron, cadmium, chromium, nickel, mercury, vanadium and reactive radical species	Neurological disorders (Minamata disease), Skin lesions, cardiovascular diseases, blackening of teeth and tongue	[18]
Tannery wastewater	Hexavalent chromium (Cr [VI]), chlorinated phenols, vegetable and synthetic tannins, azo dyes, phthalates, antimony (Sb), Pb, selenium (Se), mercury (Hg), polychlorinated biphenyls (PCBs), sulfonated oils, and formaldehyde resins	Environmental pollution and occupational hazards	[19]

### Paper industry effluent

Wastewater disposal is a significant social and environmental issue in the paper industry, as global paper consumption is increasing and unsustainable, especially in Africa, Asia, and India [20]. A significant global concern is the utilisation of raw materials for papermaking, and owing to the extensive usage of forests globally, biodiversity survival has impact on climate change [21]. India’s paper industry, which generates about 2.6% of the globe’s paper annually, comprises 859 units, thus ranking it the 15th largest production centre in the world. Most paper is produced in key states like Maharashtra, Gujarat, West Bengal, Andhra Pradesh, Odisha, and Karnataka. In contrast, comparatively significant quantities are produced in Tamil Nadu, Haryana, Assam, Bihar, Kerala, and Uttar Pradesh [20]. Significant volumes of wastewater are produced by the paper and pulp manufacturing sector as an outcome of pulping, bleaching, papermaking, and wood chip processing. The lignin-rich effluent known as black liquor is produced when lignin and hemicelluloses break down. The employment of dangerous chemicals, including hydrogen peroxide, calcium oxide, hydrochloric acid, and chlorine during the bleaching process, increases the toxicity levels in the resulting effluents [22]. The effluent comprises organic debris, wood extracts, cellulose, lignin, suspended particles, diterpene alcohols, and chlorinated resin acids [23]. The paper and pulp industry effluent is treated using the following types of processes. Filtration, flotation, and sedimentation all constitute part of the primary treatment. Biological techniques such as aerated lagoons and activated sludge are prime instances of secondary treatment methods. Anaerobic processes include acetogenesis, hydrolysis, methanogenesis, acidogenesis, as well as sequential treatment. Reverse osmosis, membrane separation, ozonisation, precipitation, coagulation, nanofiltration, microfiltration, and ultrafiltration are all components of the tertiary process [24]. It has been demonstrated that the submerged polyvinylidene fluoride membrane bioreactor (MBR) can remove 79% of pigment and 73% of COD from pulp and paper industry effluent. Recently, a distinctive anaerobic moving bed biofilm reactor-microbial fuel cell (MBBR-MFC) was created to simultaneously treat wastewater from paper mills and produce bioelectricity [25]. These innovative methods demonstrated 65.6% COD elimination efficiency and excellent bioelectricity efficiency (power distribution: 94.5 mW/m^2^; internal resistance: 35.7 Ω) [26]. 

### Petrochemical derivative effluent

Petroleum refineries are the most polluting industry, Oily wastewater contains dangerous substances like petroleum hydrocarbons, polyaromatic hydrocarbons, and phenol. Petroleum refineries produce refined products like lubricants, fuels, and liquefied petroleum gas, with effluent varying significantly based on crude oil properties, plant structure, and process designs, causing adverse impacts on humans, animals, and aquatic plants [27]. The petroleum refinery wastewater treatment facility comprises of primary treatment, which includes gravity separation, coagulation-flocculation equalizationzvdsa tank, dissolved air flotation (DAF), induced air floatation (IAF), and screening, to reduce oil, grease, turbidity, total suspended solids (TSS), and large floating debris from the wastewater [27]. Conventional approaches for treating petroleum refinery wastewater (PRWW) include membrane separation technology, coagulation, flotation, and biological treatment. One of the most significant barriers to adopting biological processes to treat PRWW is toxic, recalcitrant contaminants from hydrocarbon sources, such as naphthenic acids. Activated sludge units are usually treated biologically after mechanical and physicochemical procedures. Anaerobic baffled reactors (ABR), membrane bioreactors (MBR), granular sludge beds (EGSB), sequence batch reactors (SBR), and up-flow anaerobic sludge blankets (UASB) are among prominent technologies. Current literature suggests that contemporary wastewater management facilities are progressively integrating advanced oxidation techniques, including: Fenton and photo-Fenton reactions, ozonation, photocatalytic oxidation, and electro-oxidation. These facilities, which incorporate heterogeneous photocatalytic oxidations, are also capable of more optimal removal of contaminants and produce diminished volumes of secondary byproducts which also require treatment [28].

### Textile dyeing effluent

The textile industry employs a range of devices, techniques, and raw materials to produce the required qualities. The primary source of effluent formation is the extensive use of water during preparation or chemical methods. The amount of effluent generated by the printing, dyeing, and finishing processes increases; 45% is produced during the preparatory stage, 33% during the dyeing process, and 22% at the finishing stage [29]. In the preliminary stage of textile effluent treatment, pollutants such as oil, organic waste, grease, grit, and suspended particles are removed. Subsequently, the wastewater is filtered to remove coarse suspended particles such as rags, yarn, lint, fabric fragments, and fibers. Primary treatment like sedimentation and flocculation removes coarse materials, excess oil and grease, and suspended particles from wastewater to prepare them for biological treatment [30]. Secondary treatment effectively eliminates increased BOD, oil, and phenol from wastewater, and stabilizes it by removing or reducing color, dissolved, and colloidal organic components, unlike sedimentation [29]. This method involves the use of a trickling filter and activated sludge process. Due to the rapid rate of breakdown and capacity to adsorb toxic organic compounds, activated sludge is an effective approach for detoxifying effluent from textile dyeing [31] Tertiary treatment is required for textile effluents to eliminate impurities and render them suitable for reuse. The adsorption of organic colorants and membrane filtration of dissolved particulates are typical procedures. Nearly 99% of contaminants are eliminated by using ozone or other oxidizing agents. The integrated dyeing wastewater treatment plant employs a combined Fenton oxidation and membrane bioreactor (MBR) process to reduce organic carbon and color by 40% and 69% after 35 min of reaction under ideal Fenton oxidation conditions [29].

### Tannery effluent

The partial chemical removal process of tanning raw hides or skin into leather products results in wastewater from the tannery industry that contains a multitude of chemicals. Among these elements are proteins, hairs, chromium salts, tannins, sulphides, acids, alkalis, chlorides, solvents, colors, auxiliaries, etc. Typically, the hide or skins do not completely immobilize the chemicals employed in tanning procedures, resulting in a basic, dark brown waste with an elevated level of BOD, COD, TDS, phenolics, chromium, high pH, and a pungent odor [32]. One well-known technique for eliminating buoyant colloidal fragments from wastewater is flocculation/coagulation [33]. Over the past two decades, various technologies such as microfiltration, ultrafiltration, nanofiltration, and reverse osmosis have been developed to treat contaminated water. Membrane filtration, which employs pressure, flow, and concentration gradients to simultaneously remove contaminants, has emerged as a focal point for the treatment of tannery wastewater (TWW) [32]. To break down, mineralize, and purify contaminants in wastewater samples, sophisticated oxidation techniques are employed. For organic pollutants that decompose readily, biological methods work effectively; nevertheless, they are inadequate for refractory organic compounds. This problem has been addressed by the development of advanced oxidation processes (AOPs) that include photocatalytic treatment, photo-electro catalytic oxidation, ozone treatment, and wet air oxidation method [34, 35].

## Types of nano-engineered catalysts for pollutant removal

**Fig. 2:**
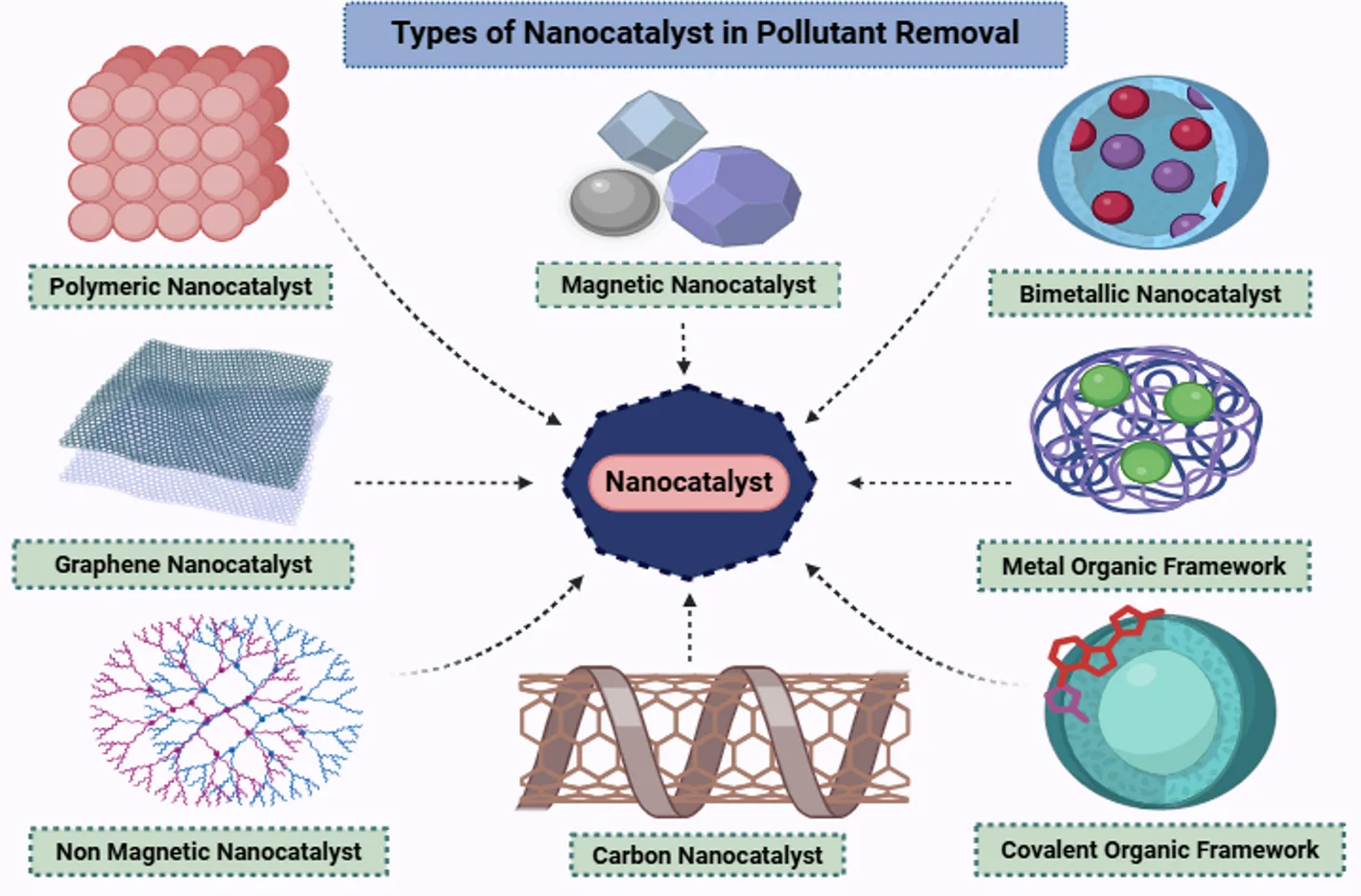
Types of Nanocatalyst involved in pollutant removal

Nano-engineered catalysts comprise an array of customized advanced materials which act to speed up the breakdown, conversion, or elimination of contaminants from effluents resulting from various industrial processes. These catalysts (metal-based nanostructures, carbon nanomaterials, bimetallics, MOFs/COFs, enzyme-mimetic nanozymes) possess distinct physical and chemical properties leading to higher adsorption and redox activity as well as superior catalytic selectivity. The major classes of nanocatalysts which facilitate the removal of contaminants and their functional roles and environmental applications, are illustrated in Fig. 2.

### Metal-based nanocatalysts

Most nano catalysts are composed of metallic nanoparticles and semiconducting materials, which have significant potential activity against highly concentrated organic pollutants, bacteria, viruses, and radioactive contaminants [36]. Due to their easy retention and reusability, magnetic nanoparticles make a desirable nano-catalyst [37]. Fe_2_O_3_ nanoparticles are employed as antimicrobials, Fenton-based, and photocatalysts. They can oxidize and break down organic contaminants when mixed with hydrogen peroxide. When sprayed with H_2_O_2_, they can also harm phenol and ethylene glycol at room temperature, even in the absence of UV or other visible irradiation sources [1]. The reduction of heavy metal ions has been explored by affixing ligands or chelating agents to the surfaces of functional magnetic nanoparticles. The alterations enhance nanoparticles with magnetic properties more suitable for widespread water treatment applications by increasing their selectivity along with binding capacity for targeted heavy metals [38]. The ability of nonmagnetic nanoparticles to separate and adsorb contaminants from water is the centre of significant research. These include zerovalent iron nanoparticles (ZVINPs) and silver nanoparticles (AgNPs), which are crucial for heavy metal cleanup because of their remarkable adsorption and catalytic capabilities. The nanoparticles can be modified by adding coatings and functional groups to increase their selectivity for contaminants. They also enable metal ion binding and reduction processes due to their huge surface areas and number of active sites [39].Zerovalent iron nanoparticles, or ZVINPs, have gained an immense amount of scientific interest because of their ability to effectively reduce a range of heavy metal ions through redox reaction. Recent studies have demonstrated that ZVINPs efficiently absorb metal ions on their surface, transferring electrons to produce insoluble hydroxides of metals or oxides [40]. Yang and colleagues eliminated heavy metals from water using NZVI assisted by biochar derived from maize stalks. Researchers achieved the most significant adsorption rates for Pb (II), Cu (II), and zinc (II), respectively, of 195.1, 161.9, and 109.7 mg/g in six hours [41]. A previous study demonstrated that, that hexavalent chromium-contaminated soil achieved a 100% immobilisation efficacy of Cr (VI) when 8 g kilograms^− 1^ of biochar-supported non-zero valent ion (NZVI )had been utilised for 15 days [10].

Metal and metal-oxide nanoparticles like Fe, Fe₃O₄, TiO₂, and MnO₂ have multifaceted approaches to removing pollutants, including direct transfer of electrons (i.e. reductive transformation) and heterogeneous catalytic oxidation (i.e. oxidation of an activation intermediate) and radical generation (e.g. Iron species Fenton-like reactions). In addition, these metal nanoparticles have magnetic properties, which allows them to be easily separated after treatment [42].

### Carbon-based nanocatalysts

Carbon-based nanomaterials and carbon-based nanoparticles, like carbon nanotubes (CNTs), have special properties like the capacity to absorb organic and inorganic pollutants and their accessibility, which allows for chemical or physical modification [1].

Functionalising carbon nanotubes (CNTs) with various inorganic or organic components enhances their adsorption capacity and selectivity towards heavy metal contaminants. They can efficiently remove lead, arsenic, chromium, mercury, and other contaminants through coprecipitation with certain metal oxides, such as zirconium and iron. Due to the unique one-dimensional structure of the carbon nanotubes, metal ions migrate into the inner pore spaces, resulting in a rapid absorption rate as well as high removal efficiency [43]. CNTs can be produced using methods such as chemical vapour deposition, laser ablation, and arc discharge. The adsorption capacity of CNTs is greatly influenced by the methods employed to create them using various reacting agents and catalysts [10].

Heavy metal remediation commonly uses graphene-based nanomaterials, including graphene oxide and reduced graphene oxide. Graphene oxide nanosheets were used to absorb cadmium and cobalt from water [1]. Extended chemical processes (like electrochemistry, sol-gel, micro-emulsion, as well as hydrothermal processes) and simple chemical processes (like oxidation by chemicals and deposition) are the main chemical methods for functionalising graphene for ecological purposes. By utilising these methods, functional groups can be added to carbon nanomaterials to enable them to carry out an array of functions [44]. For heavy metal ions, including nickel, lead, and cadmium, graphene oxide (GO) composites with various functional groups have exceptional adsorption capacities. Due to the unique structure of graphene-based nanocomposites, contaminants can be removed from aqueous solutions quickly and accurately, offering potential solutions to reduce heavy metal pollution [45].

Carbon nanomaterials (e.g. graphene, CNTs, activated carbon, carbon dots) use primarily adsorption and catalytic activity of electrons. They can enhance photocatalysis and electrochemical degradation, as well as ROS formation, by charge transfer and/or metal addition [46].

### Bi-metallic catalyst

The increased activity, selectivity, and stability of bimetallic catalysts, which result in the synergistic effects of the interaction of the two different metal components, have been studied. Catalytic inactivity of a surface component usually results in aggregate effects.The essential metal component is diluted to various atom groups or aggregates using this surface element. Smaller ensemble sizes are also useful since they promote the intended reaction while preventing undesirable and non-selective ones. Co-precipitation is one of the most feasible ways to create bimetallic catalysts with a significant metal weight-to-volume ratio. This process guarantees tiny particle sizes with a metal concentration of at least 70% [47]. A hybrid of Na_2_Mn_4_O_13_ and MO_3_ was coated with Au-Pd NPs to produce incredibly stable and potent nano-hybrid bimetallic catalysts. A comparison of the catalytic activity of treatments with Methylene Blue, Brilliant Green, and Safranine dyes was conducted. The highest catalytic activity was achieved at a mass ratio of 1:1 Au/Pd and a calcination temperature at 400 °C [48]. Fe-Cu/zeolite was additionally employed to treat bisphenol A (BPA), with a catalyst dosage of 3 g/L as well as a Cu/Fe ratio of 1:2 at pH 5, a 87% degradation rate was achieved in 120 min. The bimetallic catalyst’s advantages included low chemical costs, high reactive oxygen species production, simplicity of preparation, and simple operation for high-end applications [49].

Due to synergistic effects, like improved stability as well as tunable adsorption energies and enhanced electron transfer, multimetallic nanoparticles (e.g. Pd-Fe, Au-Ag) and bimetallic nanoparticles are advantageous. One of these metals can oxidatively activate an oxidant, whereas the other can lower activation barriers by enhancing the pollutants electron transfer and/or binding [50].

### Metal-organic frameworks (MOFs)

Porous materials having large surface areas and pore topologies that can be altered by metallic ions or clusters organised with organic ligands are known as metal-organic frameworks, or MOFs. According to recent research, MOFs can effectively remove heavy metals from the environment due to their photocatalytic reduction capabilities. The organic and inorganic components, respectively, stand in for the metal clusters and organic linkers [44].

The organic linkers in MOFs are extremely diverse and can be found in a variety of combinations. There are over 20,000 MOFs known to exist because of the diverse combinations of metals and organic linkers. The MOFs and the additional nanoparticles (NPs) within the MOF cavities work in conjunction to provide several advantages that outweigh their disadvantages. For example, better catalysis efficiency is attained, which is not attainable via the separate components [51]. In the photocatalytic degradation of organic pollutants, MOFs have demonstrated great promise. MOFs made of light-absorbing organic ligands and metal ions have light-absorbing antennas that act as unique semiconductor quantum dots. In response to these discoveries, researchers have developed a range of light-responsive MOFs for the sequestration of CO_2_, the production of hydrogen as well, the selective synthesis of organic matter, the breakdown of organic contaminants, and the eradication of bacteria from wastewater [52].

#### Covalent organic framework (COFs)

Covalent organic frameworks, or COFs, exhibit exceptional properties in several interdisciplinary domains, such as electricity, sorption, gas storage, and catalysis. They are easily modified with functional groups and have distinct, crystal-porous structures. Recently authors have evaluated the capacity of COFs to eliminate radionuclides and found that they could selectively adsorb radionuclides if their pore sizes, skeletons, and sites of action could be thoroughly designed and constructed accordingly [53]. Spectral methods and computer simulations were used to investigate the interaction between contaminants and COF. COF-based nanocomposites can photocatalyze the breakdown of organic materials in the presence of natural light and adsorb organic molecules by precipitation, sorption, as well as catalytic reduction/degradation. The researchers discovered that COF-based catalysts effectively produce electron/hole pairs and absorb visible light, enabling heavy metal ions to form precipitates on COFs, or COF-based materials, by decreasing strong valence metals to low valence metals [54].

Metal and organic framework/ covalent organic framework structures (MOFs/COFs) have the ability to adsorb, concentrate, and catalytically transform pollutants due to their large surface area, tunable active sites (i.e. metal nodes, organic linkers) and functionalities as heterogeneous catalysts. They can also be photocatalysts when using light-absorbing linkers, as well as supports for metal nanoparticles [55].

### Enzyme-mimetic nanocatalysts (Nanozymes)

Nanozymes as a class of functioning nanomaterials have the ability to mimic natural enzymes and catalyze the four main types of biological reactions, which include activities of oxidoreductase, hydrolase, isomerase, and lyase. They include supramolecules, biomolecules, inorganic nanomaterials, metal nanoparticles, metal organic frameworks, covalent organic frameworks, and nano-carbons. The catalytic performance of nano-carbon nanozymes is substantially affected by their size and shape [56]. Since Fe_3_O_4_ MNPs could be efficiently synthesised from inexpensive and plentiful precursors and have enzymatic activity equivalent to that of peroxidases, they are frequently utilised for the oxidative degradation of metallic substrates and wastewater treatment. They are also investigated for the degradation of environmental pollutants [57]. Nanozymes’ ability to mimic laccase was used to degrade phenolic contaminants. Excellent catalytic efficiency and recyclability were demonstrated by CH-Cu nanoparticles, which were bioinspired by the structure of laccase and the electron transfer pathway. They demonstrated outstanding stability at high pH, temperatures, and salt concentrations and maintained 82% catalytic activity after 12 cycles [58].

In comparison to native enzymes, nanozymes are far more stable and robust. These nanozymes mimic natural enzymes, such as peroxidases and oxidases, which catalyze substrate oxidation and generation of ROS [59].

### Photocatalytic nanoparticles and nanocomposites

Photocatalytic pollutants such as TiO₂, ZnO, g-C₃N₄, and doped semiconductors can generate charge carriers through photocatalytic activations such as solar radiation/fluorescent light and hollow cathode lamps. TiO₂, ZnO, and g-C₃N₄ can produce very reactive and aggressive oxidation species, and oxidizing and changing to ashes various types of contaminants such as dyes, pharmaceuticals, phenol, and numerous industrial contaminants [60]. Metal and non-metal oxides or semiconductors, doping, and forming heterojunctions, multifunctional or photocatalytic nanocomposite metals, such as nanocomposite-metal semiconductors, carbon nanocomposite- and polymer-supported photocatalysts can increase photocatalytic activity of nanocomposite metals to pollutants. Nanostructures or photocatalytic nanocomposites, photostatic, and nanoplates, or high surface area nanostructures, or MOFs, mesoporous oxide frameworks can improve charge separations and enhanced turnovers of photocatalysts. A multifunctional photocatalyst, high surface area nanocomposite photocatalysts, Nanocomposite photocatalysts to micropollutants, and photocatalysts to pollutants in industrial effluents [61].

Table 2 summarizes the performance of various nanocatalysts in pollutant removal, detailing their synthesis methods, target contaminants, and removal efficiencies.

**Table 2:** Performance of nanocatalysts in pollutant removal

Nano catalyst	Synthesis method	Target contaminant	Removal efficiency	Reference
Bi₂WO₆ (Bismuth tungstate)	Hydrothermal synthesis	Ciprofloxacin, tetracycline, sulfamethoxazole	> 90% photocatalytic degradation under visible light	[62]
Bi₂WO₆/g-C₃N₄ heterojunction	Hydrothermal + calcination	Rhodamine B	~ 98% degradation within 60–90 min under visible light	[63]
Bi₂WO₆/Ag composite	Photodeposition	Methylene blue	~ 99% degradation with enhanced charge separation	[64]
Bi₂WO₆/TiO₂ composite	Sol-gel + hydrothermal	Organic dyes	> 95% degradation due to improved visible-light activity	[65]
Bi₂WO₆ (Bismuth tungstate)	Hydrothermal synthesis	Rhodamine B	94% degradation within 60 min under visible-light irradiation	[66]
Mesoporous Bi₂WO₆	Template-assisted hydrothermal synthesis	Phenol	97% degradation within 60 min under visible light	[67]
Bi₂WO₆–TiO₂ heterostructure	Hydrothermal synthesis	Rhodamine B / Phenol	Enhanced photocatalytic degradation compared with pristine Bi₂WO₆ and TiO₂ due to improved charge separation	[68]
Hybrid nanocomposites, N-TiO_2_/graphene/Ag, N-TiO_2_/graphene/Au	Photo-electro catalytic and photo electroctalysis	Diazinon	81.1% for N-TiO2/G/Ag and 76.7% for N-TiO2/G/Au were observed at pH = 6 in 60 min	[69]
zinc oxide nanoparticles (ZnO-NPs)	Green-synthesis with date palm waste	Methylene blue and Eosin yellow dyes	Degradation efficiency is 90%	[70]
Mn-Al	Ball Milled	Brilliant Green	100% removal from wastewater	[71]
ZnO/rGO	Chemical deposition	Metalaxyl (MX)	90% removal of Metalaxyl	[72]
Ce & Zn doped CuO nanocatalyst	Co-precipitation	Methyl Orange	81% Degradation of Methyl Orange	[73]
α-MnO_2_/TiO_2_	One step facile hydrothermal	Coomassie brilliant blue R-250	98.35% degradation in 30 min	[74]
CuO Nanorods	Chemical synthesis	Textile dye Reactive Black 5 (RB-5)	Degradation of RB-5 was achieved around 98% within 300 min	[75]
Ag-Mo/CuO NPs	Biogenic synthesis	Methylene blue (MB) dye	Photocatalytic degradation is 99%	[76]
Iron oxide nanoparticles	Green synthesis through *Hylocereus undatus* extract	Mentanil Yellow and Orange Ⅱ	95% degradation of Mentanil Yellow and 67% of orange Ⅱ	[77]
CeO_2_-carbon nanotubes	Catalytic Ozonization	Phenol	96% in 60 min	[78]
Multiwalled carbon nanotubes (MWCNTS)	Hydrothermal reaction	Methylene Blue	99% degradation in 10 min	[79]
CuO-Go/TiO_2_	Hydrothermal Method	2-chlorophneol	86% removal in 210 min	[80]
TiO_2_/ MgO	Chemical Precipitation	Methyl Orange, Alizarin Red	83.2% degradation for methyl orange and 43.8% for alizarin red	[81]
Nanosilica particles	Chemical Precipitation	Methyl orange	100% degradation in 30 min for 10 mg/L pollutant concentration	[82]
Ag-doped ZnO	Co-precipitation	Methylene Blue (MB)	Degradation of MB ~ 65–95%	[83]
Zn-Co-layered double hydroxide	Pyrolysis	Gemifloxacin	92.7% of Gemifloxacin was degraded through photocatalysis	[84]
STA-12 (Fe, Mn)	Photo-Fenton oxidation	Rhodamine B and methylene blue (MB)	93% degradation under 40 min of natural light	[85]

## Advances in synthesis and fabrication of nano-catalysts

### Green synthesis approaches

In comparison to traditional methods, green synthesis, which uses algae, bacteria, fungi, plant extracts, and yeasts, is safer, non-toxic, environmentally benign, and comparatively more cost-effective. The efficiency of producing nanomaterials could be increased, and several areas could undergo a revolution owing to this approach [86].

The distinctive characteristics of nanomaterials, such as their size at the nanoscale, surface morphology, and interactions with other materials, are drawing more attention to them [87]. Specifically, top-down and bottom-up methods are used to synthesize them [56]. Nanoparticles, produced through a single-step bio-reduction process, offer enhanced stability and suitable diameters. Green synthesis methodology uses extracts from plants, bacteria, fungi, and animals, providing an environment for nano-sized catalysts without the need for surfactants, capping agents, or templates [88].

#### Microbial-mediated synthesis

Non-pathogenic bacteria have produced graphite carbon incorporated with iron/nickel (Fe/Ni), the NCs, which are remarkably resilient at higher temperatures. As the carbon source, *Escherichia coli* treated with Fe/Ni produced a highly propagated Fe/Ni alloy embedded in graphitic carbon. Given their capacity to catalyse tar cracking, improve redox processes, and inhibit carbon deposition, these NPs were employed [89]. Rhodium/N-functionalized carbon-encapsulated nickel composites were produced biogenically by pyrolyzing bamboo fungus with pre-absorbed nickel nitrate. These composites catalyse the hydrolytic dehydrogenation of ammonia borane and are magnetically retrievable. They can produce hydrogen for up to five cycles without diminishing their catalytic activity [90].

Using algae and fungi for biogenic synthesis has developed as an environmentally friendly method of making nanoparticles of metals and metal oxides that have adjustable sizes, shapes, and surface compositions. Polysaccharides, proteins, pigments, and polyphenols are abundant in micro and macro algae and serve as both reducing and stabilizing agents [91]. When algal biomass or extracts come into contact with metal salts, metal ions are reduced and nucleate into nanoparticles which end up yielding spherical or quasi-spherical particles in a matter of minutes to hours depending on the pH, temperature, and concentration. This method has been widely reviewed and has been shown to produce nanoparticles of Au, Ag, TiO_2_, ZnO, and various other metals that have the potential for use as antimicrobials or catalysts [92].

Filamentous and yeast fungi can also synthesize nanoparticles using both intracellular and extracellular methods. With extracellular synthesis, an enzyme such as a reductase or oxidase, as well as a peptide, are secreted to reduce the metal ions and control the particles, making collection of the end product easier [93]. With the intracellular method, metal ions are taken up by the fungal cells and the reduction and nucleation of the metal ions happens within the cell which can create unique morphologies of the particles. Because fungi can produce high quantities of biomass quickly, they are a very promising option for scale up as well [94].

#### Plant-mediated synthesis

In contrast to microorganism-based synthesis, which involves heating the reaction medium or culture medium, metallic NPs are primarily synthesized at ambient temperature using plant extracts. For large-scale production, plant-mediated synthesis is therefore preferred over microbes [95]. The petiole extracts of the *Piper betle* were used in the sustainable production of magnetic pallidum through decreased graphene oxide. The resultant material demonstrated catalytic activity for three continuous cycles of usage and was proved to be a magnetically recoverable heterogeneous hybrid NC for Suzuki–Miyaura conjugation reactions of biphenyl compounds [96]. A floral extract from *Helleborus niger* was used to biogenically synthesize copper nanoparticles mounted on Fe_3_O_4_. The phytochemicals in the extract were shown to keep the produced nanoparticles viable. These nanoparticles were also utilized to create a variety of pyrano (3,2-c) chromene compounds that could be recovered magnetically and employed eight times without exhibiting any discernible decrease in catalytic activity [97].

### Chemical and physical synthesis methods

**Fig. 3:**
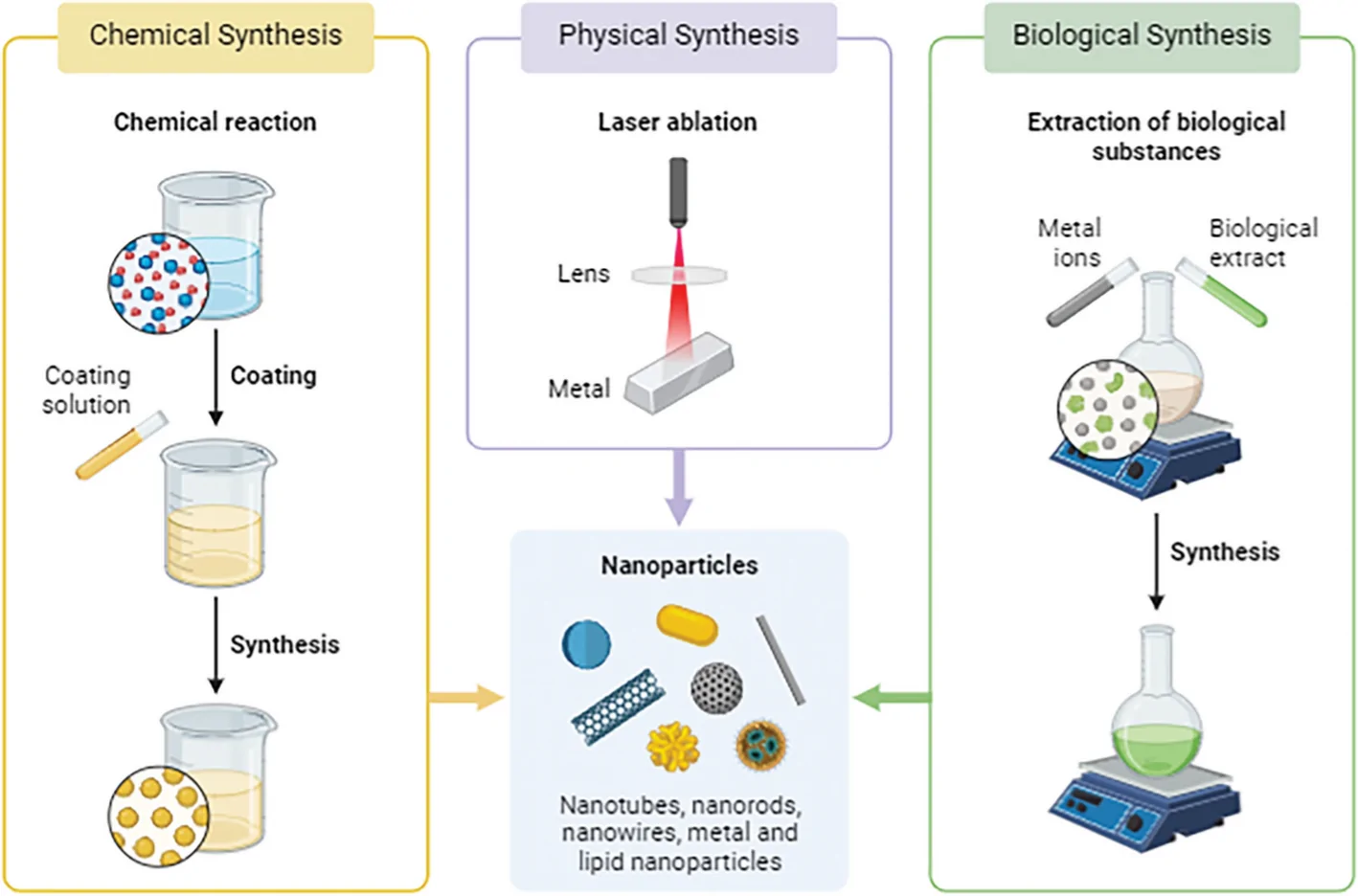
Different types of synthesis methods of nanoparticles [99]

An approach of chemical synthesis that creates nanoparticles by heating and gelling a sol-gel synthesis, or a colloidal fluid made of nanoparticles. The process, which involves producing nanoparticles at elevated temperatures and pressures, can be improved and energy consumption decreased by employing temperature reduction and moderate reactions [98]. As illustrated in Fig. 3, nanoparticles can be synthesized through both chemical, physical methods, and biological methods.

#### Sol-gel method

The sol-gel process, which transforms the precursor substance into a colloidal material (sol) and then gels the reaction mixture, is a popular approach for producing metal oxide nanostructures. Centrifugation or sedimentation are used in this process to separate the liquid and solid phases. The product offers good control over size, homogeneity, purity, and low temperatures which is also stable. Following the combination of precursors, a homogenous sol is formed, transformed into a gel when the solvent is eliminated, and then the resulting gel is dried. The equivalent metal alkoxide is a typical molecular precursor that can be dissolved in water or alcohol and heated to form a gel [100]. The most prevalent technique for producing nanostructured matrix materials, including tungsten oxide, cerium oxide, and NiO nanoparticles, is sol-gel synthesis [101].

#### Solvothermal/ Hydrothermal

One technique for creating crystalline materials is hydrothermal melting, which is mostly utilised to create composed and controlled single crystals at high pressure and temperature. Considering its wide control over morphology, ability to reduce particle size, and appropriate post-processing techniques, it is appropriate for high-purity MO nanoparticles and large-scale production. However, for optimum performance, it requires a sterilising chamber and long response times [102]. To produce nanostructures, a precipitating agent, like NaOH, is incorporated with a soluble in water, metal precursor solution, sealed with a Teflon-lined autoclave, maintained at a steady temperature of 80–200 °C for 1–20 h, followed by sterilised and annealing. The solvent used in this technique may have a significant impact on the nanostructures’ size, shape, and crystalline structure [103].

#### Chemical vapor deposition (CVD)

Chemical vapour deposition (CVD) is a green chemistry technique that involves heating a precursor before injecting it into a reaction chamber to produce an active gaseous reactant. The precursors utilised in this process are non-toxic, volatile, and non-pyrophoric. When the substrate surface is exposed to volatile precursors, chemical reactions occur that cause the deposition of desired products. Through CVD techniques, carbon nanotubes and metal oxide/nanostructures have been created, including ZnO nanocrystal powders, nanoparticles of iron dioxide, Fe_3_O_4_ nanoflowers, and TiO_2_ nanowires [17].

#### Mechanical ball milling

Materials must be mixed at a 2:1 ratio, containing solid iron or carbide balls in a container, to be converted into nanoparticles. The material squeezes between the spheres and the wall because of the container’s fast rotation. The size and production rate of nanomaterials are influenced by the rotation speed and milling time. A variety of milling container types are employed, including vibrating ball mills, high-energy ball mills, planetary ball mills, attrition ball mills, and low energy rolling mills [104].

### Surface modification and functionalization strategies

Surface modification or functionalisation techniques include coating with inorganic layers like silica, metals, metal oxide, or sulphide, or grafting with molecules like biomolecules, polymers, or molecules of small organic compounds [105]. The process of functionalisation is used to alter a material’s physicochemical characteristics, of magnetic nanoparticles. It includes defect generation, surface modification, and polymer functionalisation, which are productive in a variety of applications because of their distinct qualities, like their dimensions, form, and crystalline phase [106]. When carbon nano absorbents (CNAs) are functionalised, their functional groups interact with heavy metals, influencing adsorption mechanisms such as precipitation, ion exchange, surface complexation, electrostatic contact, and physical adsorption. Furthermore, the functionalisation of CNAs leads to enhanced electrostatic interactions between the adsorbent surface and cations, which oversee adsorption selectivity. Modifying CNAs entails doping heteroatoms on the C-surface for adding various functional groups. Additional advantages of functionalisation include increased electrostatic stability, which facilitates the removal of carbon compounds. Altering the chemistry of the carbon surface is vital for the development of novel and efficient adsorbents and their various applications. The type of precursor material, chemical agents, and modification process are some of the factors influencing functionalisation [107]. K_2_FeO_4_ was used as an oxidizer in a one-pot oxidation process to create CNT-based composites. This oxidant is widely utilized as an effective reagent to create hybrid composites of carbon nanotubes and magnetic nanoparticles. Excellent Cu (II) ion adsorption onto a hybrid CNT composite was demonstrated by the results, which also showed that the manufactured composite had the highest regeneration potential following adsorption [108].

## Applications in industrial effluent treatment

**Fig. 4:**
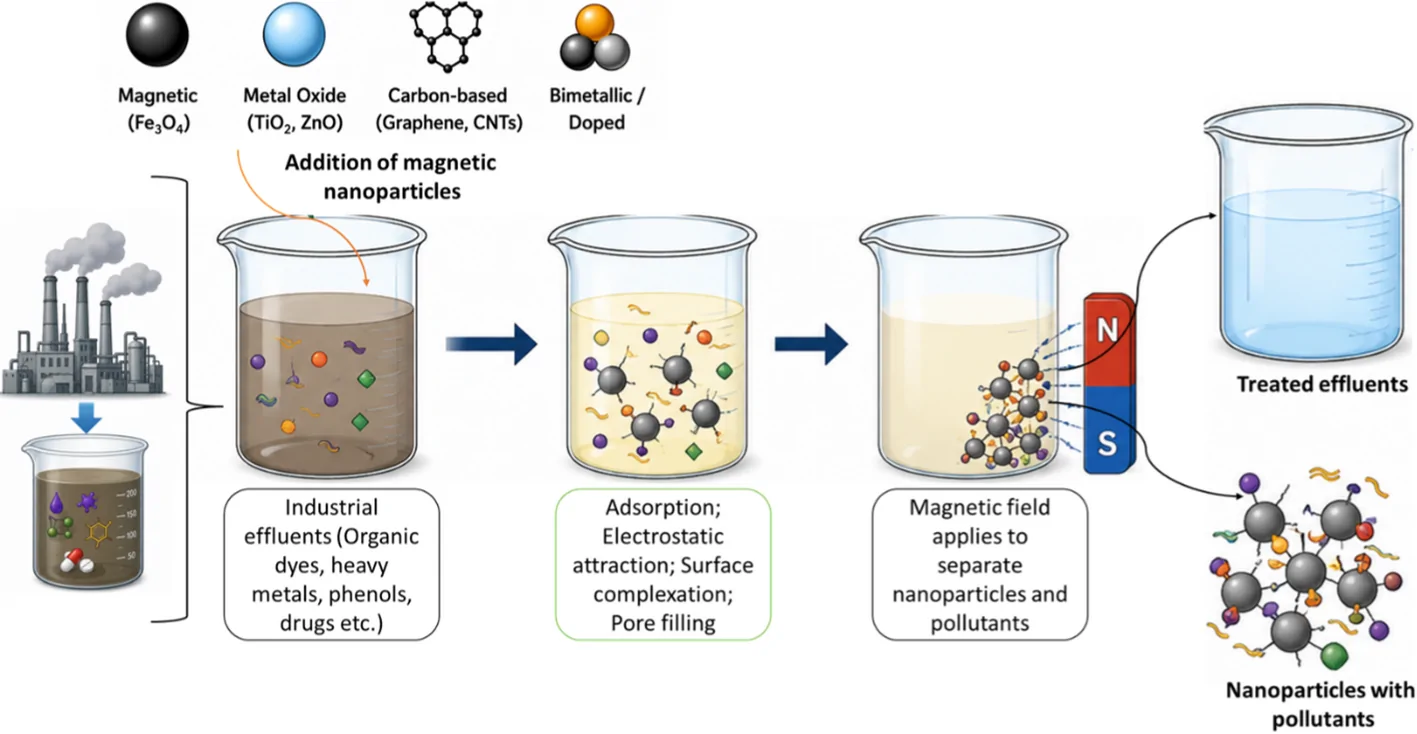
Application of nanoparticles in the treatment of Industrial Effluent

Untreated industrial effluents are a danger to the environment and public health because they are a source of a wide range of contaminants. These contaminants include dyes, heavy metals, pharmaceuticals, pesticides, hydrocarbons, and other persistent organic pollutants. Coagulation, activated sludge processing, and chemical oxidation are traditional treatment methods. There are many advantages to the use of nano-engineered catalysts, especially with these traditional treatment methods’ challenges. These nano-engineered catalysts have high surface reactivity, they are efficient with adsorption, and they are tunable with their physicochemical properties and their catalytic efficiencies are high. These nano-engineered catalysts are especially useful in advanced oxidation processes, redox transforms, and selective degradations of various contaminants. Examples of wastewater rich in these contaminants include but are not limited to, tannery, textile, petrochemical, pharmaceutical, and paper industries. These catalysts are becoming the most advanced and efficient methods to manage and treat wastewater in industrial settings. Figure 4 demonstrates the application of nanoparticles in the treatment of industrial effluent.

### Heavy metal removal

Plants and animals are negatively affected by heavy metals, which are five times denser than water and include mercury, arsenic, cadmium, lead, and copper. These materials have a shiny surface, conduct heat, and conduct current. Although they might vary in concentration, they are found throughout nature. Concerns about them are growing every day as they become dangerous when concentrations beyond safe limits [17].

The elimination of Cd (II) contaminants from wastewater and soil water has been regarded as a crucial procedure that improves the safety and quality of the water since the existence of trace levels of this pollutant in water can have devastating impacts on the ecosystem and living things [109]. Through an adsorption method between pH 4 and 8, Tasharrofi used the CoFe_2_O_4_ NPs/FAU-ZLs nanocomposite to remove Cd (II) from contaminated groundwater. In the nanocomposite, sodium zeolite (Naz) provided bigger ionic-exchange sites for CoFe_2_O_4_ NP access, and it eliminated not only Cd (II) but also Pb (II), Cr (III), Hg (II), and Ni (II) metals from tainted groundwater sources [110].

Al-Kadhi extracted Cd (II) from wastewater with 90% efficiency using a NiO NPs/MW-CNTs nanocomposite. The negative charge that was generated from the nanocomposite allowed for greater removal, and the adsorbent dosage was adjusted for pH 7–8. On the other hand, metal hydroxide, Cd(OH)_2_, precipitated when the adsorption rate decreased at pH > 8.0 due to increasing basicity. The charged Cd (II) and the two electrons on the oxygen atoms of NiO interacted electrostatically and van der Waals to occupy the Cd (II) with hexagonal arranged carbon atoms from MW-CNTs [111].

Prabhu and colleagues created a nanocomposite of Fe_3_O_4_ NPs and activated carbon matrix made from bone debris (Fe_3_O_4_ NPs/AC), which is employed as a nano-sorbent to remove Cr (VI) from wastewater. Physical adsorption of Cr (VI) on the Fe_3_O_4_ NPs/AC nanocomposite surface (adsorbent dose of 5 g/L) was found to be effective, with a concentration of 27.86 mg/g at pH 3. The adsorption of Cr (VI) followed a pseudo-first-order kinetic response and showed the Freundlich isotherm model. Furthermore, after treating the adsorbent for one hour, the elimination of Cr (VI) at pH 3 approached 100% [112].

**Fig. 5:**
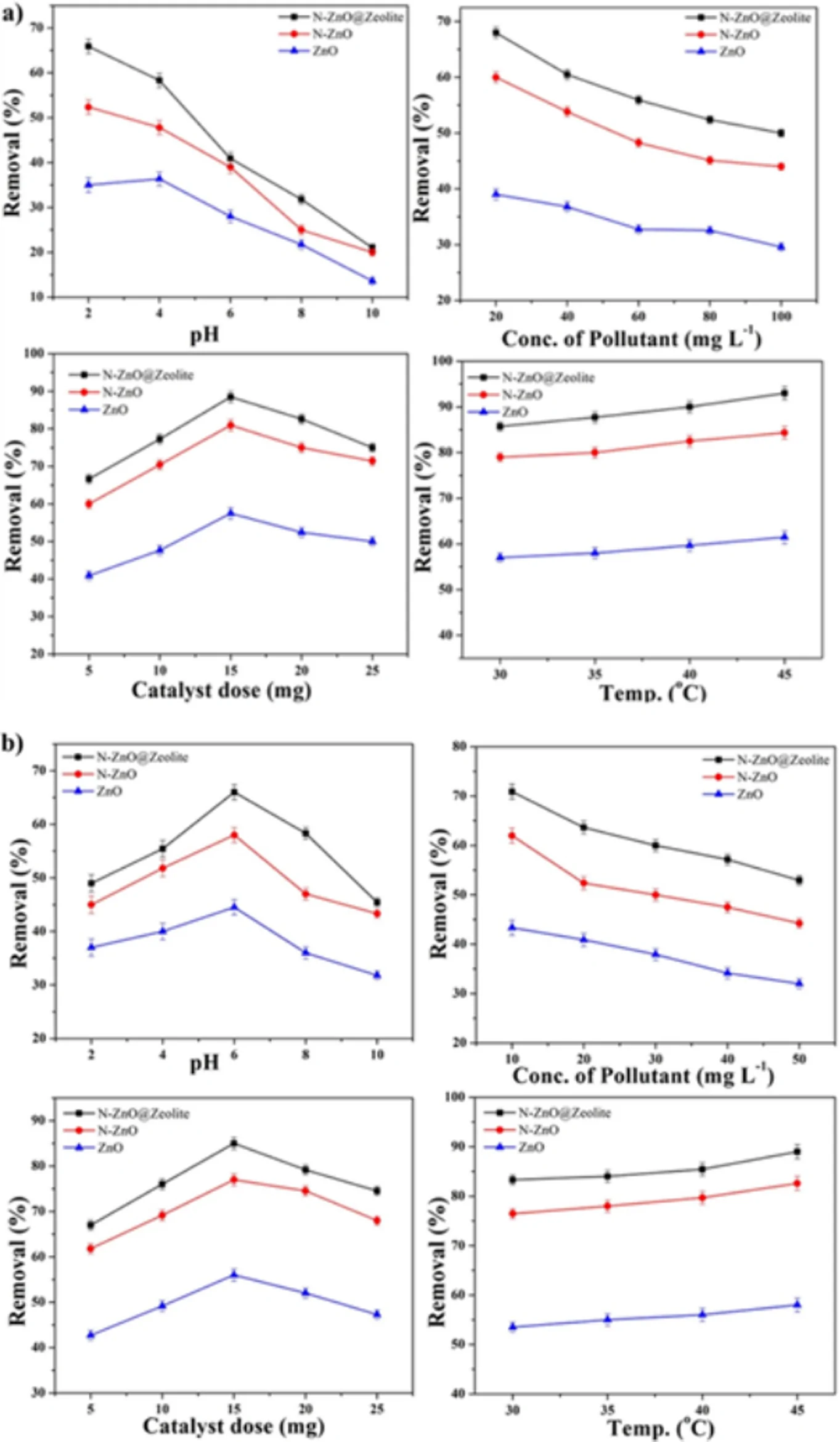
Removal of (**a**) Cr⁶⁺ and (**b**) Cd²⁺ using ZnO, N-doped ZnO, and N-ZnO@Zeolite nanocomposites. Note: All experiments were performed in triplicate (*n* = 3) to estimate error bars. Reproduced with permission from Vipin et al. (2023) © Elsevier 2023

Figure 5 illustrates whether the Cr^6+^ and Cd^2+^ reduction percentages were impacted by the adsorbent dose. With adsorbent doses up to 15 mg, the reduction efficiency first rises before falling. The lowest uptake was 5 mg, while the highest removal was achieved at 15 mg. The removal process is temperature-dependent, as evidenced by the increasing adsorption efficiency with solution temperature. As the temperature rose, the viscosity of the solution decreased, which improved ion mobility [113].

*Oryza sativa*(OSL),*Saccharum ravannae*(SRL),*and Saccharum officinarum* (SOL) leaves were used to prepare SiO_2_NPs (SNPs) [114]. The produced nanoparticles were used as adsorbents to extract heavy metal ions Pb (II) and Cu (II) from artificial wastewater. SRL SNPs had adsorption capacities of 140.06 mg/g and 149.25 mg/g for Pb(II) and Cu(II), SOL SNPs had adsorption capacities of 338.55 mg/g and 179.45 mg/g, and OSL SNPs had adsorption capacities of 334.7 mg/g and 274.02 mg/g [114]. A recent study outlined the biosynthesis of silica-based zinc oxide nanocomposites that may be used as an adsorbent to remove the heavy metal ions nickel, cadmium, and copper from synthetic water. Concerning silica-based zinc oxide nanocomposites, the corresponding adsorption rates for nickel, cadmium, and copper ions were 32.53, 32.10, and 30.98 mg/g [115].

### Degradation of organic pollutants

Conventional wastewater treatment techniques seldom eradicate agricultural pesticides, which are a serious hazard affecting people and the environment. The prospective use of nano-catalysts along with nano-composite adsorption approaches in wastewater treatment is being investigated because of their high removal and breakdown rates [116]. Nanocomposite materials have increased applications due to their synthesis, enhanced reactivity, and volume/area correlations. Adsorption techniques, particularly those using nanomaterials, are often used to eliminate pesticides from wastewater due to their ease of use, low energy consumption, and specificity for organic contaminants [17].

**Fig. 6:**
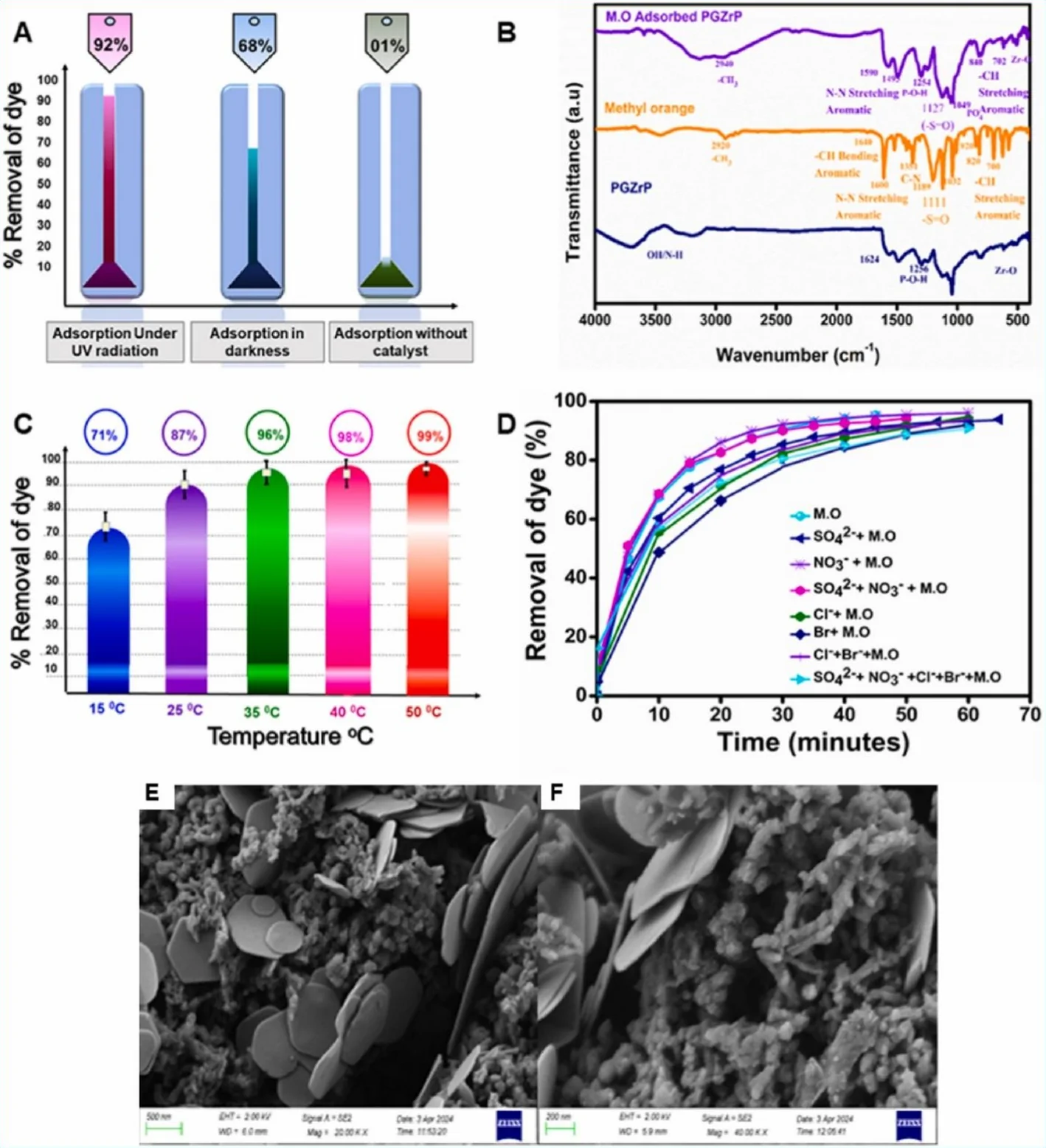
(**A**) Effect of UV radiation on the percentage removal of dye. (**B**) FT-IR spectra of MO, PGZrP, and MO-adsorbed PGZrP. (**C**) Effect of temperature on dye removal efficiency. (**D**) Influence of interfering cations on the percentage removal of MO dye. (E & F) Scanning electron microscopic images of PGZrP after MO adsorption. Reproduced with permission from [87] © Elsevier 2024

Heterojunctions Cu_2_V_2_O_7_/Cu_3_V_2_O_8_/g-C_3_N_4_ (CVCs) were created by some research group using a reheating preparation technique [117]. The thermal etching method produced a larger surface area. The CVCs demonstrated superior photocatalysis and adsorption capabilities. It was discovered that CVC-2, which contains 2 weight% of Cu_2_V_2_O_7_/Cu_3_V_2_O_8_, demonstrated greater levels for the removal of various dyes and antibiotics. Under visible light irradiation, adsorption and photocatalysis performed well to remove 96.2% of methylene blue (MB), 97.3% of rhodamine B (RhB), 83% of ciprofloxacin (CIP), 86% of tetracycline (TC), and 80.5% of oxytetracycline (OTC) [118]. Pomegranate peel extract was employed for generating the bimetallic nano zero-valent iron (nZVI)-Cu NPs for remediation purposes; a tetracycline removal of 72 ± 0.5% (with an initial tetracycline concentration of 10 mg L^− 1^) has been reported with a nZVI-Cu concentration of 750 mg L^− 1^ at pH 7. Bentonite-supported composites have been used to increase tetracycline removal and address colloidal instability; these composites showed an increase in removal with a significantly lower nanoparticle loading [119]. By combining the advantages of graphene oxide, zirconium phosphate, and polyaniline, some researchers developed a novel composite material for the removal of methyl orange (MO) dye [120]. The adsorption tests in this investigation were conducted both in dark mode and under UV light. The adsorption efficiency for the batches that were exposed to UV light and dark mode was found to be 92% and 68%, respectively (Fig. 6).

**Fig. 7:**
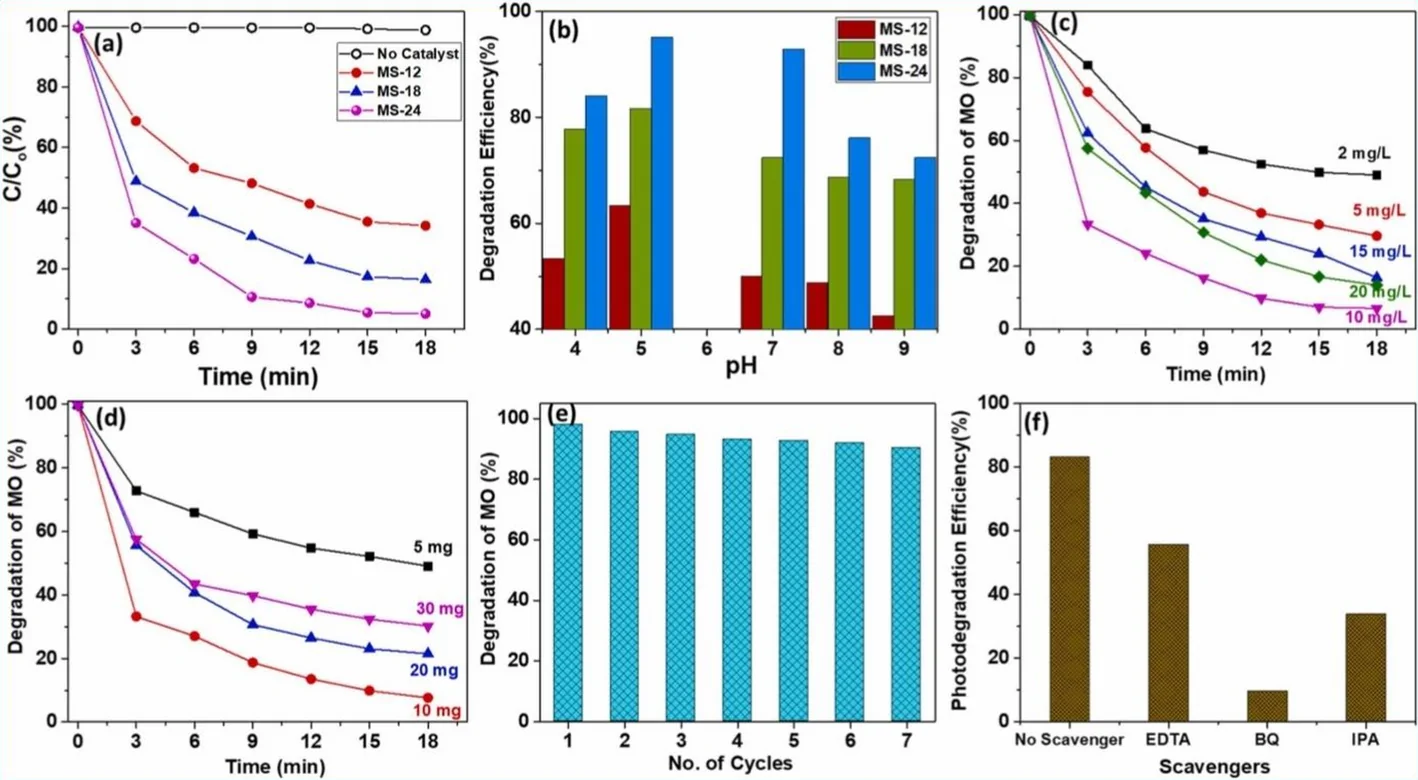
(**a**) Photocatalytic degradation performance of various photocatalysts under visible light irradiation. (**b**) Photodegradation efficiency at different pH values. (**c**) Effect of initial dye concentration on degradation efficiency. (**d**) Influence of catalyst concentration on photocatalytic activity. (**e**) Reusability of the catalyst for MO degradation over seven consecutive cycles. (**f**) Photodegradation efficiency of MS-24 in the presence of different reactive species scavengers. Reproduced with permission from [89] © Elsevier 2024

A recent report examined the photodegradation capacity of MoS2 nanostructures to degrade Methyl orange (MO) in response to light [89]. As shown in Fig. 7, the photolysis of MO was negligible in the absence of a photocatalyst, as demonstrated by the UV-visible absorption study. Nevertheless, MS-24 exhibited the highest photocatalytic efficiency among MS-12 and MS-18, achieving 94% of photocatalytic dye degradation in a mere 18 min. MS-24’s exceptional recycling stability was demonstrated by the significant impact of the dye solution’s pH on its properties. The study also examined the impact of reactive oxygen species on the photodecomposition of MO. The results indicated that the MO is likely to undergo a transition to an ionic quinone structure in acidic conditions, which will lead to a more favorable photodegradation response. The study also investigated the impact of varying MS-24 concentrations on the photodecomposition of MO. The decomposition rate of MO increased as the catalyst concentration was increased; however, the degradation was reduced by a further increase to 30 mg as a result of light scattering and inadequate light penetration in the reaction mixture. The reusability and stability of MS-24 were examined, and it exhibited exceptional photocatalytic efficiency for a maximum of seven repeated cycles [121].

The removal of the pesticide Malathion, an organophosphate having carcinogenic properties, from water was examined by some research group employing iron oxide nanoparticles (Fe_3_O_4_ NPs) as a catalyst in the photocatalysis process [122]. The response surface method was implemented to eliminate malathion. Several factors, including pH, Fe_3_O_4_ NP content, and exposure duration, affect the elimination of malathion. In terms of eliminating malathion from aqueous media, the study found that 0.4 g/L of Fe_3_O_4_ NPs, an approximate one-hour contact duration, and a pH of 5 using ultraviolet radiation produced the best results (82% was eliminated) [122]. TiO_2_ nanoparticles (NPs) with photocatalytic capabilities were produced by Alhalili and Smiri [123] using an aqueous extract of *Aloe vera* leaves. After calcination, the NPs changed into spheres with particle sizes of 23 ± 2 nm (1-dimensional) and 83 ± 5 nm (2-dimensional). Remazol Red Brilliant F_3_B (RR180) has been removed from aqueous solutions using the nanoparticles. The NPs exhibited greater photodegradation (100%) of the RR180 dye after one hour of exposure to visible light, whereas those with an 83 ± 5 nm diameter demonstrated an efficiency of 100% for up to two hours [123].

### Reduction of toxic inorganic compounds

Water contains nitrate, which can be converted to nitrite by stomach bacteria and cancer-causing N-nitrosamine chemicals. Congenital impairments and stomach cancer can result from elevated nitrate levels. Non-structured carbohydrates can be accumulated by nitrogen-deficient plants, and nitrate, nitrite, and ammonia can be harmful to humans, animals, and plants. Phosphate and nitrates can be eliminated from water systems using the Nano-Fe (OH)3/Clinoptilolite technique. Following the dissolution of FeSO4.7H2O in methanol and water, clinoptilolite is added. Despite a 1:2 Fe/Clinoptilolite ratio producing increased effectiveness, the Fe/Clinoptilolite ratio increases the efficiency of Nano-Fe (OH)3/Clinoptilolite. A 5:1 ratio between Fe and Clinoptilolite yields greater efficiency [124].Rice husk ash-derived silica nanoparticles are used to remove nitrate from the wastewater. The amount and efficiency of nitrate removal depend on several factors, such as pH, adsorbent mass, adsorbate concentration, and contact time. At pH 6, the silica nanoparticles adsorption capability of 14.22 mg per gram of nitrate particles is attained. The adsorption capacity of silica nanoparticles is increased when their surface functional groups are altered (To et al., 2020). The green synthesis of iron oxide nanoparticles (NPs) dispersed on zeolite by eucalyptus leaf extracts was used to remove both ammonia and phosphate from aqueous solutions over a specific period. The generated material removed 99.8% of PO_4_^3−^ and 43.3% of NH_4_^+^ at a primary concentration of 10 mg L^− 1^ for each of the two co-existing ions. The produced material’s maximum adsorption capacity for NH4 + and PO_4_^3−^ was 3.47 and 38.91 mg g^− 1^, respectively, after optimising evaluations [125].

**Fig. 8:**
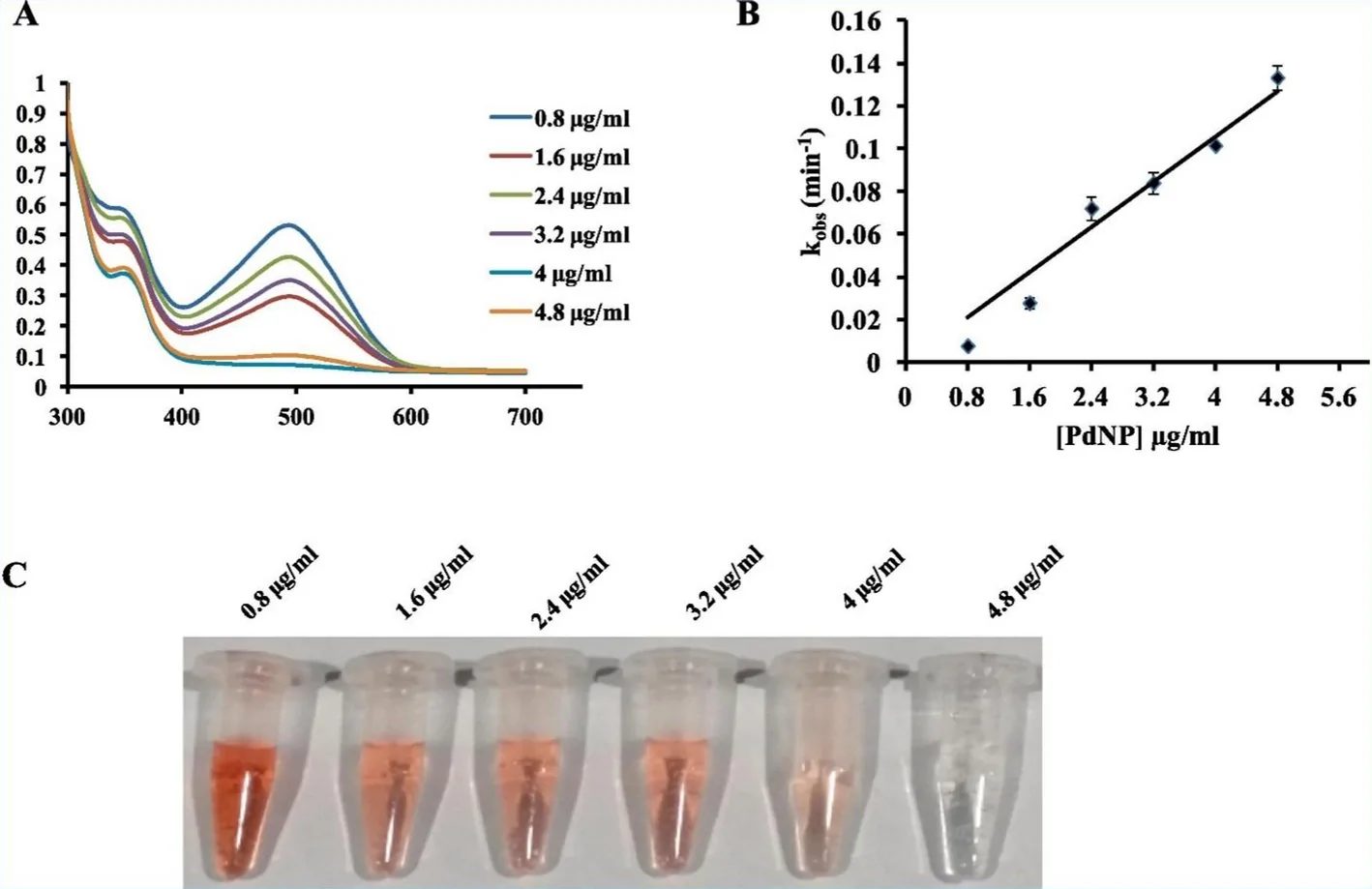
(**A**) UV–Visible spectra showing Congo Red (CR) degradation at varying concentrations of palladium nanoparticles, with a fixed dye concentration (150 µM) and NaBH₄ (10 mM) as the reducing agent. (**B**) Degradation rate constants as a function of palladium nanoparticle concentration. (**C**) Visual images depicting CR degradation at different nanoparticle concentrations. Reproduced with permission from [94] © Elsevier 2024

A recent study developed catechol-generated palladium nanoparticles (PdNPs) to selectively remove congo red (CR) dye from wastewater. The degradation process occurs within 10 min, and the recycled catalyst exhibits 60% efficiency even after the third cycle. The degraded products show benzidine formation, indicating the reductive cleavage of the dye’s azo linkage [94]. The adsorption capacity of the nanoparticle catalyst is limited, and the efficiency decreases with increasing dye concentration. The degradation rate constant also decreases with higher dye concentrations (Fig. 8).

### Removal of emerging contaminants

Endocrine-disrupting chemicals (EDCs) are emerging contaminants that can damage the endocrine system and aquatic ecology. Magnetic nanoparticles (MNPs) can separate EDCs from water using an external magnetic field. Magnetite, a cheap, readily available MNP, is used in most literature research due to its high surface area and ability to bind analytes and transfer energy quickly, facilitating rapid equilibrium. Alongside magnetite (Fe_3_O_4_) nanoparticles, which are inexpensive and simple to obtain, are other metallic oxide nanoparticles (MOPs), such as oxides of zinc, copper, and nickel. Remarkable EDC recovery rates (> 90%) were demonstrated in studies employing MNPs with diameters ranging from 10 to 80 nm, low concentrations (0.071–20 mg/mL), and short adsorption intervals (0.5–15 min). These variables help in the development of water treatment techniques. The use of magnetic nanoparticles in the removal of endocrine disruptors from wastewater is a promising model as they are cheap and readily available [126].

**Fig. 9:**
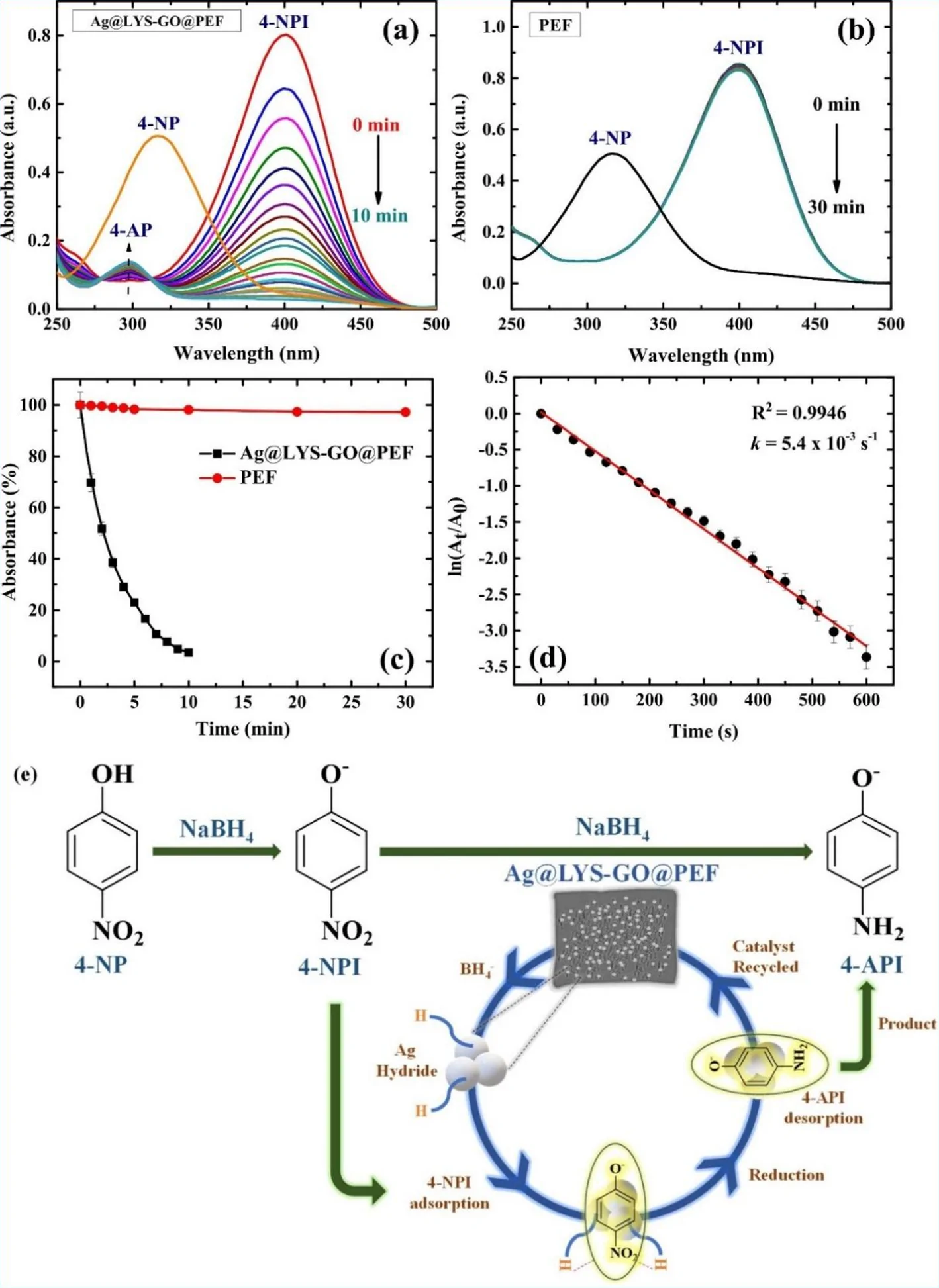
(**a**) UV-Vis spectra showing the reduction of 4-nitrophenol (4-NP) using the Ag@LYS-GO@PEF catalyst. (**b**) UV-Vis spectra of 4-NP reduction using pristine PEF as a control. (**c**) Plot of percentage absorbance versus time for the reduction process. (**d**) Variation of ln(At/A₀) as a function of time, indicating the reaction kinetics. (**e**) Proposed mechanism for the catalytic reduction of 4-NP. Reproduced with permission from [127] © Elsevier 2024

Some research group examined the catalytic efficacy of an Ag@LYSGO@PEF substrate catalyst in the NaBH4-based reduction of 4-nitrophenolate ion (4-NPI). The conversion of 4-NP to 4-NPI is facilitated by the maximal UV absorption of the aqueous solution of 4-NP. Over time, the absorbance of 4-NP was considerably reduced by the application of the Ag@LYS-GO@PEF substrate, resulting in a gradual fading of the dark yellow color and a peak at 401 nm to colorless [127]. The Ag@LYS-GO@PEF catalyst’s robustness was underscored by the fact that the conversion efficiency of 4-NP remained consistent even after 10 repetitive cycles (Fig. 9).

Composites containing graphene or reduced graphene oxide (rGO) were the subject of extensive research among titanium oxide-based photocatalysts. The TiO_2_x/rGO nanocomposite was created using a hydrothermal-calcination process in which TiO_2_ was self-doped by Ti^3+^ and oxygen vacancies (Ti^3+/^Ov). Improved visible light harvesting was made possible by the existence of Ti^3+/^O within the oxide lattice, and an effective charge separation was made possible by the chemical interactions (Ti-O-C) between TiO_2_x and rGO. Additionally, the combination of TiO_2_x and rGO enhanced the adsorption of organic contaminants by preventing TiO_2_x particle agglomeration. TiO_2_x/rGO demonstrated a 6.16, 2.92, as well as 2.55-fold increased rate of reactivity for BPA degradation in contrast to conventional TiO_2_, TiO_2_/rGO, and TiO_2_x [128].

A study by Yan et al. (2023) investigated the significance of nanoparticle aggregate interactions at the nanoscale decomposition of MPs by iron oxide nanoparticles via analyzing particle aggregate microstructures and tracking dynamic light scattering properties. Fe3O4 nanoparticles with particle sizes ranging from 100 to 1000 nm were produced by coprecipitation, and their efficacy in removing MPs was investigated. The subsequent effects on the removal effectiveness of microplastics were determined by examining the processes of particle aggregation under different salinity and pH conditions. The results showed that Fe3O4 nanoparticles could remove 83.1% to 92.9% of the MPs in an hour [129]. The naturally existing algae *C. vulgaris* were utilized by Peng and colleagues to create inexpensive biohybrid microrobots with Fe_3_O_4_ NPs applied to their surface. Rather than exhibiting the uncontrolled Brownian motion that plain algal cells displayed, the surface ornamentation enabled precise activation and influence of the microrobots. Compared to static conditions (41%), the active motion of the as-prepared microrobots enabled a significant efficiency increase (up to 92%) in the removal of amino-modified polystyrene (MPs and NPs) from model suspension. The microrobots demonstrated significant potential in eliminating MPs and NPs in natural aquatic conditions [130].

## Challenges and limitations

The ability of nanomaterials to combine different properties to create multifunctional systems, like nanocomposite membranes, that allow for both particle retention and contaminant removal, is one of their most significant advantages over traditional water technologies. Functionalized materials that have NPs integrated into them or coated on their surface carry potential hazards since NPs could be discharged into the environment, where they could accumulate over time. Numerous national and international laws and regulations are being implemented to reduce the health risk. The primary technical drawback of nano-engineered water solutions is their inability to be scaled for large-scale operations and their current inability to compete with traditional treatment systems [131]. There are several obstacles associated with green synthesis techniques that should be addressed promptly to develop more effective strategies for addressing the problems that are still present. The creation of NCs with consistent sizes remains one of the foremost and most essential problems. When the NPs are manufactured on a larger scale, they tend to group, which reduces their surface area and, consequently, their catalytic ability and versatility. Therefore, to attain long-term stability, their morphology must be maintained. By giving the particles substantial matrices to sustain themselves, NC aggregation can be avoided. However, when the nano catalysts are resting on a solid substrate, caution should be exercised to avoid blocking their catalytic sites [132].

The development and broad use of green nanomaterial synthesis are hindered by challenges in materials selection, reactivity product quality, and efficiency, even though it has great promise [133]. Due to issues with sensitivity to sample matrix effects, sophisticated surface functionalisation, and the expensive and unstable nature of employing enzymes as recognition components in conjunction with a mediator system, biosensors for in vivo applications are still in their early stages [134].

For the development of safe and sustainable uses, it is essential to comprehend the ecotoxicological effects of nanomaterials on aquatic life. The creation of environmentally friendly nanomaterials, environmental monitoring, and thorough risk assessment are crucial measures to lessen their possible negative effects on aquatic ecosystems [135]. To increase their effective utility, more research is required to identify novel nanohybrids and multifunctional nanomaterials. To compare the production of green-synthesised nanomaterials with those made using traditional methods, cost-effectiveness studies must be examined. It is important to examine and assess the outcomes from genuine environmental settings, even if the effectiveness concerns and remedial performance assessment are usually created on laboratory scales, replicating the varying degrees of realistic exposure situations [131].

## Environmental risks and comparative sustainability of nanocatalysts

Recent research has shown that although nano-engineered catalysts are highly efficient in degrading pollutants, their uncontrolled release into the environment could pose severe threats to ecotoxicology. Studies focused on metal-based nano-particles such as silver, copper oxide, and zinc oxide show how they can cause oxidative stress, membrane lysis, and metabolic aberrations in aquatic Daphnia and fish. Even though carbon-based nanomaterials are considered to be less-toxic, they are able to bio-accumulate and cause shading that would otherwise render primary producers less photosynthetically active [136]. There are several factors such as agglomeration, dissolution, and changes in the surface structure that determine the reactivity, as well as the toxicity, of the nanocatalysts in wastewater treatment plants. Reports show that nano-particles associate with those in sludge flocs, and pose risks of reintroducing these particles into the environment through sludge recycling [137]. New approaches have been developed to reduce the risks described above, including surface functionalization to reduce toxicity, immobilization of the particles to stable supports to prevent leaching, magnetic sequestration to facilitate sustained elution, and the creation of self-deactivating or biodegradable nanocatalysts. Life-cycle assessments, ecotoxicity tests, and monitoring nanocatalysts in compliance with regulations will be required to ensure that risks are minimized when nanocatalysts are used in water treatment on an industrial scale [138].

Ineffective reactor configuration, insufficient catalyst longevity, poor mass transfer, and expensive synthesis methods continue to hinder the industrial scaling of nanotechnology. Those same industrial issues can be identified in academic pilot scale and full scale examples like continuous-flow photocatalytic reactors used for dye effluents, fluidized-bed catalytic systems for Cr(VI) reduction, and the use of membrane-immobilized nanocatalysts to treat wastewater from the pharmaceutical industry [139]. Of particular importance to the the practical implementation of the examples, and subsequently the texts in question, are complications related to catalyst agglomeration and the decline of the catalyst activity in the presence of complex effluent matrices and the demands for catalyst regeneration and the energy to drive the process. There are economic, engineering, and regulatory aspects of nanocatalysts used within these examples that are needed for their adoption into industrial practice and these aspects should be fully understood to improve the work [140].

The examination of the comparative properties of different types of nanocatalysts is critical to determining the best materials that can be utilized in different types of industrial wastewater case studies. Metal vs. bimetal catalysts are more efficient in the catalytic function, but can be more expensive to produce, have higher leaching and potential production cost. Tunable porous carbon nanomaterials, as well as, MOF/COFs are highly surface reusability materials that have, relative to the production of other catalysts, a less environmental and synthetic cost, but are more expensive to produce [141]. Enzyme-mimetic nanozymes, as well as, photocatalytic nanostructures, have higher operational selectivity and lower energy requirements when there is solar activation, but their long-term stability can be highly variable. Comparing and contrasting the materials in terms of cost, and/or of other efficiency of degradation, recyclability, and eco-toxicity that can be sustained can help the reviewer select materials based on the type of pollutant, the needed the extent of the pollutants to be removed, and the requirements of integrated sustainable systems [142].

The latest innovation continue to build on and expand on the the application of nano-engineered treatment systems and their integration with catalytic and bio-energy engineering. Microbial fuel cells (MFCs) alone have been shown to have their energy needs met with small volume (gram to milligram) quantities of bioavailable anodes and can provide energy or electricity to on-board catalysts. At the same time MFCs can function as bio-sensors and reactor with hybrid Ph0ttcalcytic and Microbial systems, or even support nano catalyst reactors to enhance pollutant breakdown and more selctively catalyze the indetified bioaspirated target pollutant. These intergrated or coupled systems have the capablity to support the retention and extraction of nutrients and metals from the effluent streams. Selectively this provides a a smaller carbon footprint [143, 144].

## Conclusion

The removal of various contaminants from industrial waste streams has become more efficient due to the versatility and effectiveness of nano-engineered catalysts. This review consolidates various classes of nano-engineered catalysts, particularly the latest metal and carbon-based catalysts, bimetallic catalysts, nanostructured MOFs and COFs, nanozymes, photocatalytic nanoparticles and multifunctional nanocomposites, and describes their specific characteristics, tailored surface chemistries and catalytic efficiencies. Reaction mechanisms involving photocatalytic and redox transformations, Fenton and Fenton-like reactions, adsorption-activation, and enzyme-mimicking catalysis mechanisms are integrated to describe the ability of target catalysts to degrade, reduce, or mineralize contaminants including dyes, pharmaceuticals, and other harmful compounds such as heavy metals and other contaminants of emerging concern. The catalytic stability, regeneration, real effluent applicability and other aspects necessary to bring these technologies to the industial scale are still limited. Closing these gaps with the necessary mechanistic engineering, material design, evaluation of the synthesis routes, and other aspects of engineering the system to be commercially viable will be essential for these contaminants to be developed. There is still much more work to be done to develop multifunctional catalysts.

## Data Availability

No datasets were generated or analysed during the current study.

## Abbreviations

MBR: Membrane Bioreactor
mW/m^2^: Milliwatts per square meter
g kg^-1^: grams per kilogram
Ω: Ohm
mg/g: milligrams per gram
g/L: grams per liter
UV: Ultraviolet
MBBR-MFC: Moving bed biofilm reactor-microbial fuel cell
COD: Chemical Oxygen Demand
BOD: Biological Oxygen Demand
DAF: dissolved air flotation
IAF: induced air flotation
TSS: Total suspended solids
PRWW: Petroleum Refinery Wastewater
ABR: Anaerobic Baffled Reactors
EGSB: granular sludge beds
SBR: Sequence Batch Reactors
UASB: Up-flow Anaerobic Sludge blankets
TWW: Tannery Wastewater
AOPs: Advanced Oxidation Process
MNP: Magnetic Nanoparticle
AgNPs: Silver Nanoparticles
ZVINPs: Zerovalent iron nanoparticles
NZVI: Non-zero valent ion
CNT: Carbon Nanotube
Pb (II): Lead
Cu (II): Copper
Zn (II): Zinc
Cr (VI), Cr (III): Chromium
Hg (II), Hg: Mercury
Ni (II): Nickel
Sb: Antimony
Se: Selenium
PCBs: polychlorinated biphenyls
GO: Graphene Oxide
rGO: Reduced graphene oxide
ZnO: Zinc Oxide
CuO: Copper Oxide
H_2_O_2_: Hydrogen peroxide
Fe_2_O_3_: Iron (Ⅱ) Oxide
Fe_3_O_4_: Iron (Ⅲ) Oxide
Fe_3_O_4_/AC: Iron (Ⅲ) Oxide/ Activated Carbon
SiO_2_: Silicon dioxide
TiO_2_: Titanium dioxide
CO_2_: Carbon dioxide
Na_2_Mn4O_13_: Sodium Tetra Manganese Tridecaoxide
MO_3_: Metal Oxide
Au-Pd NP: Gold-Palladium nanoparticle
Pd NP: Palladium nanoparticle
BPA: Bisphenol A
Fe-Cu: Iron-copper
MOF: Metal-Organic Framework
COF: Covalent Organic Framework
Mn-Al: Manganese-Aluminium
Fe/Ni: iron/nickel
CH-Cu: carbon–copper bond
NCs: Nanocatalysts
NiO: Nickel (II) oxide
TiO_2_: Titanium dioxide
NaOH: Sodium Hydroxide
CVD: chemical vapor deposition
CNA: carbon nano absorbents
K_2_FeO_4_: Potassium ferrate (VI)
CoFe_2_O_4_: Cobalt ferrite
FAU-ZLs: Faujasite-type zeolite layer
Naz: Sodium zeolite
NiO NPs/MW-CNTs: Nickel Oxide Nanoparticles / Multi-Walled Carbon Nanotubes
Cd(OH)_2_: Cadmium Hydroxide
NaBH_4_: Sodium borohydride
OSL: Oryza sativa
SRL: Saccharum ravannae
SOL: Saccharum officinarum
Cu_2_V_2_O_7_/Cu_3_V_2_O_8_/g-C_3_N_4_: copper vanadate compound/graphitic carbon nitride
CIP: ciprofloxacin
TC: Tetracycline
OTC: oxytetracycline
MB: Methylene blue
MR: Methyl Red
CR: Congo Red
MX: Metalaxyl (MX)
RhB: Rhodamine B
MO: Methyl Orange
RR180: Remazol Red Brilliant F3B -Azo dye
EDCs: Endocrine-disrupting chemicals
TOC: Total Organic Carbon
SDG: Sustainable Development Goal
